# Raman Scattering Enhancements Due to Super- and Subradiant Collective Plasmon Modes on Large-Area 2D-Au Arrays

**DOI:** 10.1021/acsami.5c04804

**Published:** 2025-05-22

**Authors:** Ephraim T. Mathew, Andriy E. Serebryannikov, Jacek Jenczyk, Igor Iatsunskyi, Szymon Murawka, Mikołaj Lewandowski, Maciej Wiesner

**Affiliations:** † Faculty of Physics and Astronomy, Adam Mickiewicz University, Uniwersytetu Poznańskiego 2, 61-614 Poznań, Poland; ‡ NanoBioMedical Centre, Adam Mickiewicz University, Wszechnicy Piastowskiej 3, 61-614 Poznań, Poland

**Keywords:** super- and subradiant plasmon modes, anisotropic plasmonic effect, collective plasmon mode, SERS dichroism, nonpercolated films, periodically corrugated sapphire

## Abstract

Ordered metal nanoparticle (MNP) arrays with ultrasmall interparticle gaps *S* exhibit strong enhancement of the electromagnetic (EM) near-field, known as hotspots, for surface-enhanced Raman spectroscopy (SERS) sensing. These arrays, with uniform gap sizes, are also essential for studying nonlinear Raman scattering effects and surface selection rules. Optical characterization of the fabricated large-area Au arrays with *S* ≪ *r*, where *r* is the MNP radius, revealed the excitation of hybridized-collective plasmon modes with giant EM near-field enhancement. We found that the SERS enhancement associated with a subradiant plasmon mode depends primarily on the interparticle gap distance *S*, rather than on the ordering of MNPs into arrays. However, arranging MNPs in the form of arrays influenced the far-field scattering of the super-radiant mode excited at a longer wavelength, resulting in lower but highly anisotropic SERS enhancements that depend on the far-field excitation polarization angle σ. This study on ordered MNP arrays with ultrasmall interparticle gaps *S* ≪ *r* highlights the roles of S and MNP ordering in SERS enhancement of an analyte. This understanding is pivotal for designing SERS substrates with very small interparticle gaps, as they generate a large number of intense and well-distributed SERS hotspots. Furthermore, an anisotropy-induced SERS dichroism effect was observed. Polarization-dependent SERS intensities varied based on the excitation wavelength λ_exc_ and its corresponding Stokes wavelength positions related to the excited plasmon mode. As a result, the SERS dichroism of lower-frequency Stokes-shifted peaks exhibited a cos^2^(σ) dependence, whereas higher-frequency Stokes-shifted peaks exhibited a sin^2^(σ) dependence. This observation validates the EM near-field mechanism of SERS. The fabricated large-area 2D-Au arrays meet most of the essential requirements for efficient, robust, and reliable large-area SERS sensing.

## Graphical abstract

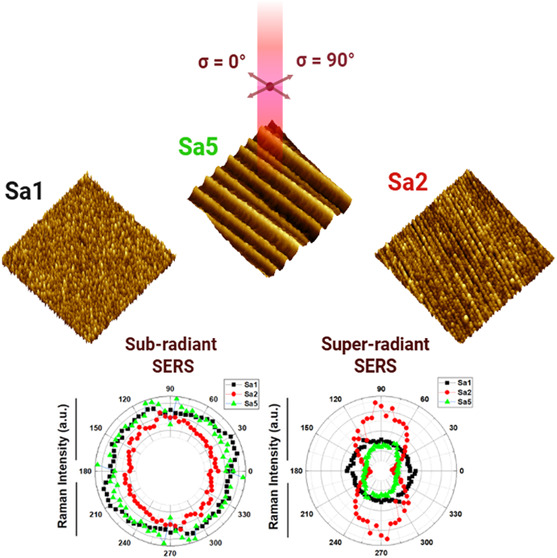

## Introduction

Surface-enhanced Raman scattering (SERS) is a phenomenon associated with the enhancement of Raman signals of analytes adsorbed on metal nanostructures. The mechanism of this enhancement involving the excitation of surface plasmons was suggested and proposed by Albrecht, Creighton, and Philpott.^1^ Importantly, the dependence of SERS intensity based on the enhanced near-field due to localized surface plasmon excitations was reported by Moskovits.^2^ Structural modulations induced on metal surfaces reorder the collective oscillation of free electrons ω_p_ in it. This results in the excitation of different types of plasmon modes (quantized collective oscillation of electrons ℏω_p_) in the metal/dielectric interface when coupled with photons (quantized electromagnetic field *hν*) under resonance conditions. These are known as plasmon polaritons.^2,3^ As in a roughened metal surface, the enhanced electromagnetic (EM) near-field due to the polaritons is focused on its rough tips. This occurs due to the localization of charges and excitation of localized surface plasmon resonance modes (LSPR) on its sharp features along the lateral cross section.^3^ Similarly, substrates decorated with MNPs of different sizes, shapes, and arrangements also exhibit tunable LSPR modes and are the most commonly used SERS substrates.^4^ This is specifically due to the high order of near-field enhancement in MNPs.^1^ Furthermore, these excited plasmon polaritons focus far-field EM spatial components of an incident electric field *E*_exc_(λ_exc_) below the diffraction limit in confined fields *E*_p_(λ_p_) within nanometer-scale modal volumes.^5^ The average magnitude of this near-field radiation, *E*_p_(λ_p_) (polarizing the Raman signal of an analyte deposited on a MNP surface), is amplified by the averaged field enhancement factor *g* according to eq 1 as follows

1 $$E_{p}\left(\lambda_{p}\right)=gE_{\mathrm{exc}}\left(\lambda_{\mathrm{exc}}\right)$$

Therefore, the analytes’ Stokes scattered field will have a strength

2 $$E_{\mathrm{Stokes}}\left(\lambda_{\mathrm{Stokes}}\right)\propto \alpha_{R}E_{p}\left(\lambda_{p}\right)\propto \alpha_{R}gE_{\mathrm{exc}}\left(\lambda_{\mathrm{exc}}\right)$$

where α_R_ is the proper combination of the components of Raman tensor.^1^

Moreover, this Raman Stokes scattered field *E*_Stokes_(λ_Stokes_) at a different wavelength when compared to *E*_p_(λ_exc_) can be further enhanced by a factor *g* ^*s*^ and will differ from *g* based on the position and full width at half-maximum (FWHM) of the excited λ_p_. Subsequently, the intensity of the emitted SERS peaks from the MNP surface can be expressed by eq 3

3 $$I_{\mathrm{SERS}}\left(\lambda_{\mathrm{exc}}\right)\propto {\left|\alpha_{R}\right|}^{2}{\left|gg^{s}\right|}^{2}I_{\mathrm{exc}}\left(\lambda_{\mathrm{exc}}\right)$$

As a result, the SERS scattering process depends on both the near-fields enhanced at *E*_p_(λ_exc_), which excites the Raman vibrations, and *E*_p_(λ_Stokes_), which enhances its Stokes-shifted radiation.^1^ Such confined near-field *E*_p_(λ_p_) on the *MNP* surface enhanced due to the polaritons can be considered as a nanooptical resonator with the frequency of λ_p_ = ℏω_p_ of the quantized collective oscillation of electrons. The intensity in an optical resonator can be expressed by the parameter *Q*/*V*, where *Q* is related to the lifetime of the plasmon (quality factor) and *V* the modal volume.^6^ This parameter is taken into account when designing and fabricating effective plasmonic substrates. Plasmonic substrates with ordered arrays and interparticle gap distance *S* ≅ λ_lspr_, where λ_lspr_ is single-particle LSPR wavelength, can support the surface lattice resonance mode at λ_slr_ due to far-field diffractive interference and exhibit high Q-factor with a narrow FWHM of its plasmon resonance peak.^7,8^ These substrates at λ_slr_ exhibit very high near-field intensities on MNP surfaces.^9^ However, the enhancement of both *E*_p_ at λ_exc_ and *E*_p_ at λ_Stokes_ cannot be achieved at λ_slr_ because of its narrow resonance peak. This may affect the feasibility of SERS sensing of analytes belonging to the same chemical group, as they emit very similar λ_Stokes_ positions for most of their Raman modes.^10–12^ For this reason, SERS substrates with a broad plasmon resonance peak and a longer lifetime (*Q*-factor) are needed. Similarly, ordered 2D-MNP arrays with interparticle separation *S ≤ r*, where *r* is the radius of the MNP are also suitable for SERS, as near-field couplings with a *S* ^–3^ dependence dominate in these structures.^13^ At interparticle gaps as small as *S ≤ r*, when another particle enabling the same field magnitude *E*_p_(λ_p_) is nearby a MNP, the region of the coupling between their two confined near-fields gives rise to an enormous near-field enhancement known as “plasmonic hotspot”. In this case, the fields *E*_p_(λ_p_) and *E*_Stokes_(λ_Stokes_) can be further enhanced by a factor *g*′ becoming *E*_p′_(λ_p′_) and *E*_Stokes‘_ (λ_Stokes’_). These hotspots (collective plasmon-polariton modes with *E*_p′_(λ_p′_)) can facilitate higher SERS enhancement as compared to a single-particle LSPR mode with *E*_p_(λ_p_), while also exhibiting a broader resonance peak.^14,15^ This interaction of confined near-fields of the arrays and their *Q*/*V* characteristics, for *N* particles in an ordered MNP system with *S ≤ r*, where *N* is the number of particles, can be described by plasmon hybridization theory.^16,17^ Although fabrication of ordered arrays with *S* ≪ *r* is commonly considered to be difficult and unachievable for structures with dimensions and period below 20 nm, by photo- and e-beam lithography,^18^ wet chemistry^19^ and colloidal suspension techniques^20^ result in poor reproducibility due to agglomeration, disorder, and particle overlaps, which leads to nonuniform interparticle gaps. The resulting nonuniform plasmonic modes excited in these gaps, with different frequencies and near-field strengths between them, further limit the feasibility of these samples for robust and reliable large-area SERS sensing,^21^ as well as studying nonlinear Raman scattering processes and surface selection rules.^22^ On the other hand, template-assisted ordering of MNPs is a widely used technique to avoid these disadvantage.^23,24^ Several groups have reported the studies of deposition and ordering of MNPs in periodic templates.^24,25^ In our research, we specifically deposited nonpercolated Au films onto both flat and corrugated transparent-sapphire dielectric templates using an e-beam evaporation method at ultrahigh vacuum (UHV) conditions (10^–10^ mbar). Such nonpercolated ultrathin films have been earlier reported to exhibit unique plasmonic properties as they support both localized and delocalized plasmon modes with enhanced scattering cross sections desirable for SERS application.^26–28^ Here, these nonpercolated Au films are self-assembled into 2D-MNP arrays with in-plane anisotropic ordering, driven by the template corrugations under optimal deposition conditions. This approach eliminates the need for a complex glancing-angle deposition technique reported in other studies.^23,29,30^ First, we have studied the conditions for ordered nucleation of these subwavelength 2D-MNP arrays with *S* ≪ *r* and analyzed its structural and optical properties using atomic force microscopy (AFM) and polarized far-field spectroscopy. Then, we focus our study on characterizing the plasmonic hybridization in it with simulated EM near-field distributions obtained at different excitation wavelengths λ_exc_ and polarization angles σ (the angle between laser beam polarization and corrugation direction), as these collective plasmon-polariton modes *E*_p′_(λ_p′_) are highly specific to light attributes such as polarization and wavelength.^31,32^ While plasmonic hybridizations in one-dimensional MNP arrays and substrates with anisotropic MNPs shape have been studied and reported,^16,33,34^ plasmonic hybridization in anisotropically ordered 2D-MNP arrays with *S* ≪ *r* and its resulting SERS enhancements have not been corroborated and extensively studied to the best of our knowledge. Further, in our previous work, we have studied the adsorbed molecule–polariton interaction on laterally continuous corrugated plasmonic surfaces by comparing the relative SERS enhancement and anisotropy of analyte Raman peaks with different polarizability tensors.^22^ This showed the dependence of the plasmon modes surface field orientations on corrugated surfaces in the enhancement of both the diagonal and nondiagonal components of a molecule polarizability tensor. In this work, we investigate SERS enhancements resulting from both super-radiant and subradiant collective plasmon modes in these ordered plasmonic arrays by analyzing their relative SERS enhancement and dichroic SERS anisotropies. We also examine their dependence on the interparticle gap distance *S*, as well as on the degree of order and disorder in the metal nanoparticles (MNPs) in the fabricated arrays. These experimental investigations and their results are significant for the design and fabrication of SERS systems with ultrasmall interparticle gap distances. Finally, we analyze the performed polarized and wavelength-scanned SERS measurements to deconvolute the dependence of both the enhanced fields *E*_p_ at λ_exc_ and *E*_Stokes_ at λ_Stokes_ by investigating the obtained SERS relative peak intensities. The conducted polarized SERS measurements exhibited enhanced, dichroic SERS signals of phenyl-thiolate analytes dependent on the type of excited plasmon mode, λ_exc_, and σ on these samples. These results confirmed the dependence of both the field enhancements *E*_p_ at λ_exc_ and *E*_Stokes_ at λ_Stokes_ on the SERS response, thus validating the SERS electromagnetic (EM) near-field mechanism. Reliable, reproducible, and efficient SERS signals were obtained due to the features of distributed hotspot and broad Stokes-shifted spectral region in the samples.

## Experimental Section

### Corrugated Sapphire Template Fabrication

Polished sapphire (α-Al_2_O_3_) single-crystal substrates with <0,1° miscut along the M-plane [101̅0] purchased from *CrysTec GmBH* were used. When annealed at high temperatures (*T* > 1400*°* C), the unstable crystallographic plane undergoes a spontaneous asymmetric faceting, leading to the appearance of periodic, corrugated structures on its surface. A Carbolite high-temperature chamber furnace at operating temperatures up to 1700 *°*C was used to anneal the substrates. Depending on the annealing time (between 10 and 24 h) and temperature (from 1450 to 1600*°* C), the period of naturally forming corrugations was optimized.^35,36^

### Au Metal Deposition in Ultrahigh Vacuum (UHV)

An electron beam evaporation system (*Telemark*/*PREVAC*) was used to deposit Au films of different thicknesses (*t* = 2, 4, 6, 7, 8, and 10 nm) onto the reconstructed corrugated surface (see Tables 1 and S1) under UHV. The deposition was carried out at very low deposition rates of 10 and 79 Å/h on the templates. For elliptically shaped Au grain nucleation, the corrugated templates were placed at the edge of the electron beam evaporation sample stage to create an off-normal deposition angle along the direction of the corrugation vector *k*_*x*_. Further, Au grain nucleation of *d* ∼ 30 nm was achieved for a deposition rate of 10 Å/h, and *d* ∼ 60 nm was achieved for 79 Å/h onto the corrugated templates.

**Table 1:** Experimental and Structural Parameters of the Fabricated Samples: Determination of Au Grain Diameter *d* Was Not Possible for Percolated and Near-Percolating Films with Connected MNP Networks as for the Au Thickness (*t* > 7.5 nm)

sample name	Sa1	Sa2	Sa3	Sa5	Sa7	Sa8	Sa10
(*P*) corrugation period [nm]	flat	30 ± 5 nm	30 ± 5 nm	150 ± 25 nm	150 ± 25 nm	240 ± 25 nm	290 ± 25 nm
(*H*) corrugation height [nm]	flat	2 ± 1 nm	2 ± 1 nm	18 ± 5 nm	18 ± 5 nm	25 ± 5 nm	30 ± 5 nm
(*t*) Au thickness [nm]	∼6 nm	∼6 nm	∼7 nm	∼6 nm	∼10 nm	∼10 nm	∼10 nm
(*d*) Au grain diameter (nm)	27 ± 2 nm	27 ± 2 nm	elliptical grain 32 ± 2 nm in the *y*-axis, 25 ± 2 nm in the *x*-axis	27 ± 2 nm	-	-	-
Au deposition rate (Å/h)	10	10	10 (Å/h) slightly off-normal deposition	10	10	10	10

### Structural and Optical Characterization by Atomic Force Microscopy, Ellipsometry, and UV–Vis spectrometry

The topography of fabricated substrates was examined using the *Bruker Dimension Icon* AFM instrument working in soft tapping mode. Scans were recorded at different places on the sample to analyze topographic details of the corrugated surface. Reflectance analysis was performed using the *SENTECH GmbH SER800* Ellipsometer at an oblique incidence angle, θ = 50*°*. The sample was rotated with a step of Δ*σ* = 20*°* (σ—an azimuthal angle between excitation polarization and the corrugation vector *k*_*x*_ as in Figure 1a) for polarized reflectance measurements. Transverse magnetic (TM) (σ = 0°) and transverse electric (TE) (σ = 90°) polarization components were collected from the samples over the spectral range of 400–1050 nm, with a scanning interval of 1 nm. All of the reflectance measurements were normalized to a reference signal collected from a mirror reflecting 99.99% of the incident wave. Polarized-transmission spectroscopy was performed using a *PerkinElmer* UV–visible spectrometer. The source and detector positions were kept constant for transmission collection at normal incidence, while the sample was rotated to adjust the angles σ = 0, 45, and 90*°*.

**Figure 1:**
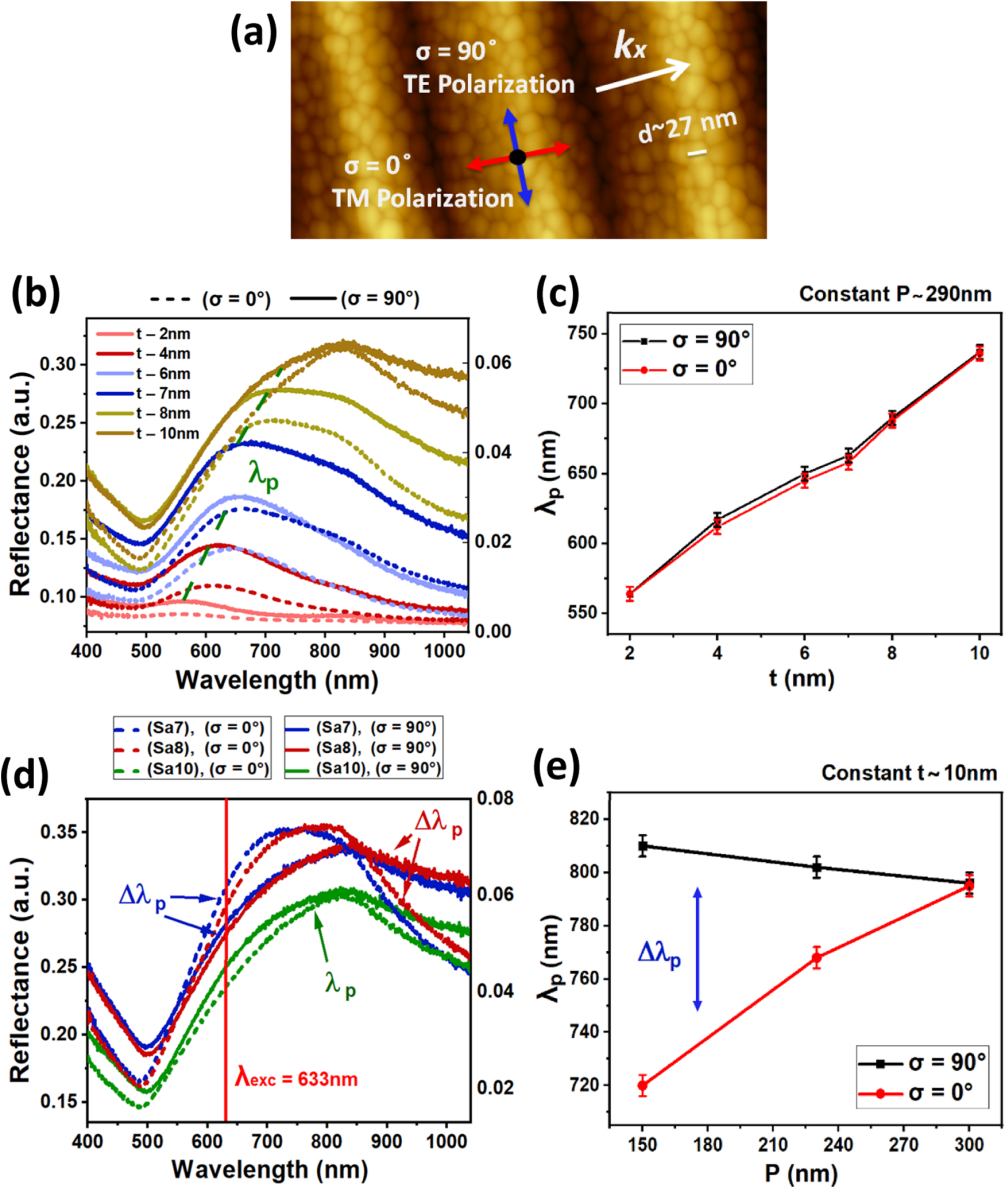
(a) AFM topography image of ∼7-nm-thick nonpercolated Au layer deposited on a corrugated template of *P* ∼ 290 nm exhibiting the randomly oriented Au grains with diameter *d* ∼ 27 nm (corrugation vector *k*_*x*_ is along σ = 0°). (b) Reflectance measured for samples Sa11–Sa16 with a constant template period *P* ∼ 290 nm deposited with varying Au thicknesses ranging from *t* ∼ 2 nm until 10 nm. (c) Quantitative comparison of λ_p_ at σ = 0 and 90°, showing very low values of Δλ_p_ for samples shown in panel (b). (d) Reflectance measured for samples Sa7, Sa8, and Sa10 (with constant *t* ∼ 10 nm, and varying *P* ∼ 150, 230, and 290 nm), exhibiting anisotropic responses between σ = 0 and 90° polarization components at decreasing corrugation *P*. (e) Quantitative comparison of λ_p_ for the samples shown in panel (d), confirming the anisotropic response due to the increasing value of Δλ_p_ for decreasing value of *P*.

### Electromagnetic Simulations Method

*CST* *Studio Suite*, commercial software based on the finite integration method, has been used for simulations. It has relatively low simulation costs and provides controllable convergence and accuracy, so that it is particularly appropriate for the studies of plasmonic and photonic periodic structures having unit cells of rather arbitrary geometry. The presented numerical results were obtained by using the frequency-domain solver with Floquet–Bloch boundary conditions and a tetrahedral mesh. Simulated Au grain arrays were homogenized in periodicity and alignment as an Au array system onto the corrugated templates. The near-field distribution simulations were obtained for a unit cell of infinite arrays, periodic along both σ = 0 and 90° directions with different interparticle distances. The power of the EM wave is assumed to be 1 W per unit cell in all of the simulations. The presented two-dimensional field distributions were obtained at the half height of Au grains, in the plane parallel to the sample surface in order to investigate the spatial distribution related to the collective plasmon mode at this plane. Maximal near-field intensity is achieved beyond the aforementioned plane. Optical constants of a 6 nm Au film provided by Yakubovsky et al.^52^ were used in the EM simulations.

### Organic Thiophenol Molecules Deposition

The fabricated plasmonic substrates were immersed in a solution of 99.8% thiophenol (*Alfa Aesar*) dissolved in 99.8% ethanol (POCH) (1:50 vol). After 10 min at room temperature, the sample was removed from the thiophenol solution and immersed in pure ethanol to remove unbonded molecules.^36^ The whole process was repeated four times to form a layer of phenyl-thiolate molecules chemisorbed to the Au surface.^37^

### SERS Measurements Instrumentation

Micro-Raman spectra were measured using a *Renishaw* *in-Via* micro-Raman system with plane-polarized lasers at λ_exc_ = 633 and 785 nm. All of the spectra were measured under a 0.7 *N*.*A.* microscopic objective within an aperture angle of 50°. At λ_exc_ = 785 nm, the laser power was 21 mW and 4 accumulations with 1 s exposure time scans were collected, while at λ_exc_ = 633 nm, the laser power was 1.6 mW and 6 accumulations with 4 s exposure time scans were collected. A spectral resolution of about 1 cm^–1^ was achieved using a 1200 and 1800 l/mm grating for the respective laser wavelengths. A *STANDA 050097* rotational step motor stage was used to perform polarized SERS measurement with a step of either Δ*σ* = 5° or Δ*σ* = 20° from σ = 0 to 180°. SERS mapping measurements were performed by scanning the laser spot at 1 μm steps to check the large-scale uniformity of the SERS signal on the samples. The SERS mapping images were processed in the Renishaw Wire software.

## Results and Discussion

When M-plane [101̅0] sapphire wafers were subjected to thermal annealing, the initially flat sapphire surface reconstructed into corrugated facets of varying periods (*P*) and heights (*H*) (Tables 1 and S1), which depend on the annealing temperature (see Sample Preparation section).^38^ Surface reconstructions with the corrugation period value of *P* ≅ 30 nm were formed on one side of the double-side-polished substrates, while larger corrugation periods with *P* ≅ 150 nm were observed on the opposite side of the wafer (see Figure S1). This formation of corrugations with different periods on both sides of the M-plane [101̅0] wafer is presumably due to the temperature gradient between the wafer surface exposed toward the air and the opposite surface side lying on the Carbolite oven sample stage. Then, Au films with *t* ∼ 2, 4, 6, 7, 8, and 10 nm were deposited at a normal incidence angle onto the templates with *P* ∼ 290 nm using a low deposition rate of 10 Å/h (see Table S1).

### Nucleation of Ordered 2D-MNP Arrays

Figure 1a shows the AFM topography image of the corrugated sample Sa14 with ∼7-nm-thick Au layer. This nonpercolated layer formed grains with diameter *d* ∼ 27 nm at a low deposition rate due to the poor wettability of Au on the sapphire surface.^39,40^ These clusters were randomly distributed due to the high surface diffusivity on the relatively wide facets with *P* ∼ 290 nm—a behavior similar to that observed on a flat surface (the corrugated templates can be regarded as short periodic elevations of facets^35^ rather than high-aspect-ratio gratings). As a result, templates with *P* ≫ *d* exhibited minimal influence on the ordering of the nucleating Au grains, resembling the behavior observed on flat sapphire templates (see Figures 1a and 2a). Furthermore, Figure 1b shows the reflectance spectra measured with an ellipsometer on templates with a constant period *P* ∼ 290 nm, deposited with varying Au thicknesses ranging from *t* ∼ 2 to 10 nm (samples Sa11–Sa16). With increasing Au thickness *t*, an incremental red shift in the plasmon wavelength (λ_p_ *)* and its peak broadening was observed. The broad peak observed at ∼830 nm is not a plasmon resonance but the sapphire template peak (see Figure S2). The plasmon wavelength shifted from λ_p_ ∼ 565 nm (FWHM ∼ 100 nm) for the sample with *t* ∼ 2 nm to λ_p_ ∼ 737 nm (FWHM > 450 nm) for the sample with *t* ∼ 10 nm at both σ = 90° (dotted lines) and σ = 0° (continuous lines) polarizations. This incremental shift of Δλ_p_ is indicated by the dotted green line in Figure 1b. The peak shift and broadening of λ_p_ can be attributed to the increasing interaction between LSPR modes of individual Au grains, as the grains become denser and closer to one another with increasing *t*. At ultralow film thickness, the fractional coverage *f* of the metal layer is *f < f*_c_ (acting as isolated grains), where *f*_c_ is the percolation threshold.^41^ Subsequently, the film approaches the percolation threshold (*f = f*_c_) with increasing *t* and undergoes a transition into a semicontinuous state, in accordance with the Volmer–Weber growth mechanism.^42^ The broadening of the λ_p_ peak is specifically caused by the increase in radiative damping rate, as dipoles excited in these randomly ordered Au grains begin to interact collectively and coherently, giving rise to a radiant mode.^28^ This behavior also depends on the number of particles *N* within the illuminated area, significantly influencing the observed plasmonic properties. Otherwise, single metal grains with a very small diameter of *d* ∼ 27 nm absorb most of the light in its λ_p_ resonance region and remain nonradiant, as described by Mie theory.^43,14^ Furthermore, the proximity region where the coupling between the confined near-fields of two neighboring Au grains gives rise to an enormous near-field enhancement—known as plasmonic hotspot—which are connected with the collective plasmon-polariton modes with *E*_p′_(λ_p′_), in line with the discussion given in the Introduction. These hotspots can facilitate higher surface-enhanced scattering when compared to the SERS enhancement due to the near-field *E*_p_(λ_p_) of a single-particle LSPR mode.^44^ Efficient light collection in the near-field along with a longer lifetime at spectral ranges of both *E*_p_(λ_p_) and *E*_Stokes_(λ_Stokes_) are essential factors for achieving enhanced SERS signal for all of the Raman peaks of the investigated analyte. This requirement will be discussed in detail in the later part of this article, in the section dedicated to SERS measurements.

**Figure 2:**
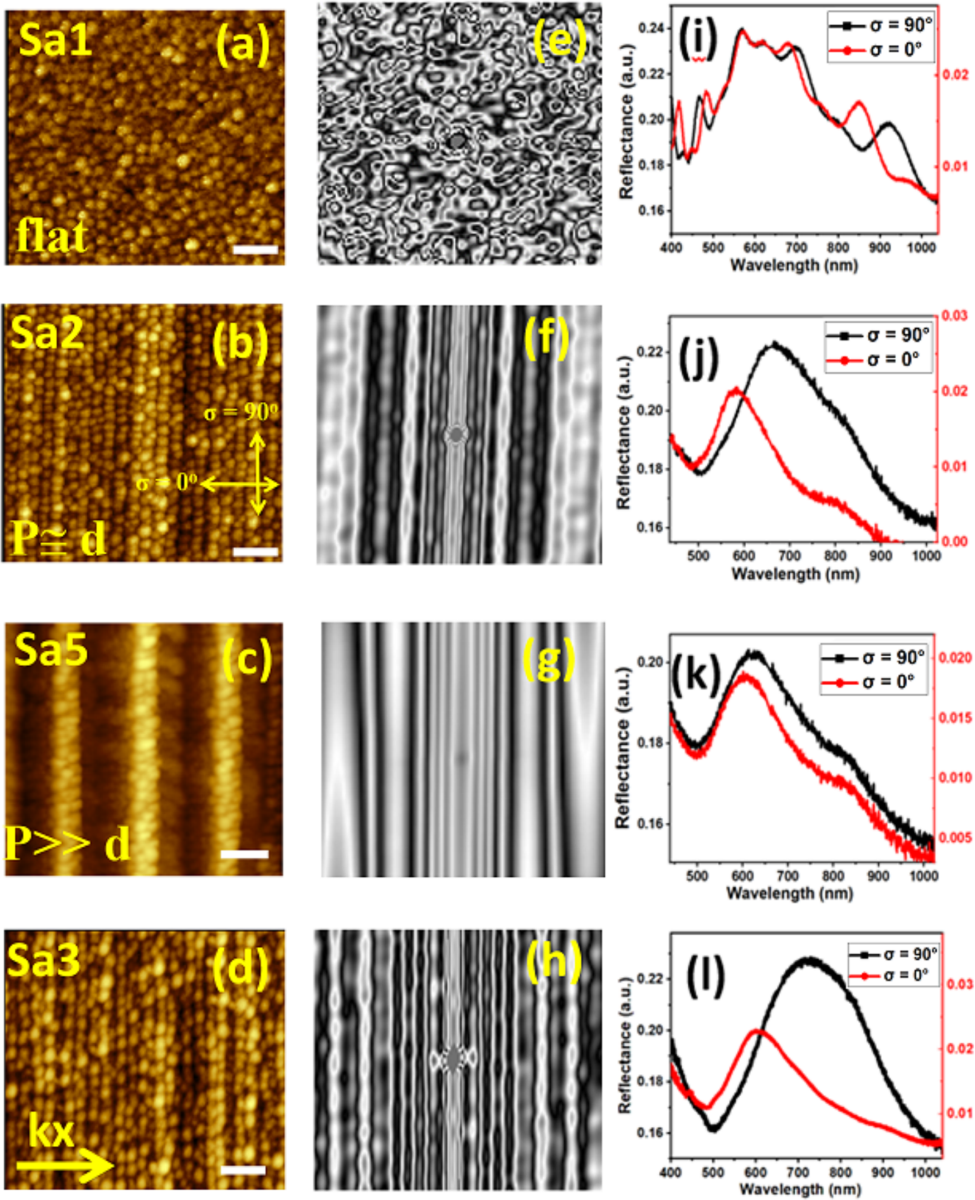
AFM topography images of samples (a) Sa1, (b) Sa2, (c) Sa5, and (d) Sa3. The white scale bars correspond to a 100 nm. Autocorrelation patterns from the AFM graphs of samples (e) Sa1, (f) Sa2, (g) Sa5, and (h) Sa3. Polarized reflectance analysis of samples (i) Sa1, (j) Sa2, (k) Sa5, and (l) Sa3 performed at the incidence angle θ = 50°.

The analysis of results obtained for samples Sa11–Sa16 in Figure 1b performed for a fixed *P* ∼ 290 nm and varying *t* reveals that Δλ_p_, which represents the difference in plasmon resonance positions between σ = 0 and 90°, remains very low and insignificant. This feature is even more evident in Figure 1c. However, analysis of the results for samples Sa7, Sa8, and Sa10 with a constant value of *t* ∼ 10 nm deposited on substrates with different *P* values exhibited an anisotropic response between σ = 0 and 90° for sample Sa7 (blue spectra) and Sa8 (red spectra) in Figure 1d. Furthermore, the data in Figure 1e provides a quantitative comparison of the λ_p_ positions at σ = 0 and 90° obtained from the plot in Figure 1d showing the increase of value of Δλ_p_ with decreasing value of *P*. This optical response with larger values of Δλ_p_ is a result of the anisotropic, in-plane ordering of nonpercolated Au films on templates with smaller period (*P*) under a linearly polarized excitation. As *P* decreases, the motion of Au adatoms toward its adjacent adsorption sites along the corrugation vector *k*_*x*_ is restricted by the corrugation that acts as a barrier, reducing the surface diffusivity along *k*_*x*_ at σ = 0°. This effect is also favored by the oblate shape of the nucleating Au grains with in-plane dimensions larger than the vertical dimension, making them very flat in the poles as spheroidal-shaped Au disks during the deposition process.^45^ This is also depicted as the optimum deposition condition when the ratio of flux of Au atoms reaching the surface and its adatom surface diffusivity cause Au nucleation density suitable for forming Au disks of *d* ≅ *P*. These thermodynamically driven kinetic processes of Au disks nucleation are further enhanced by the excellent thermal conductivity of sapphire,^39,46^ which facilitates the anisotropic in-plane ordering of Au disks as chains, even without the use of the glancing-angle deposition technique. The effect of ordering is more evident in the AFM topography images of sample Sa2 shown in Figure 2b. This sample features a nonpercolated Au layer of *t* ∼ 6 nm and exhibits template structural feature *P* ≅ *d* (where *P* ≅ 30 nm and *d* ∼ 27 nm). At a deposition rate of 10 Å/h, for which the nucleating grain diameter was *d* ∼ 27 nm, sample Sa2 exhibited anisotropic ordering of Au disks, forming ordered chain-like structures along the corrugation long axis (along σ = 90°). In contrast, for sample Sa5 with *P* ≅ 150 nm—exhibiting template features (*P* ≫ *d*)—the orientation of the disks became randomized as the value of *P* largely increased beyond *d*, as illustrated in Figure 2c. This behavior is attributed to the enhanced surface diffusion on the larger *P* facets along *k*_*x*_, as discussed earlier. Autocorrelation function in Figure 2f processed from the AFM images of sample Sa2 confirms the in-plane topographical pattern of the Au disks, resembling chains of particles. The analysis (see Figure 2f) further reveals that the disks are nucleated along *k*_*x*_ (σ = 0*°*) with an average interparticle distance *S*_1_ ≅ *P* dictated by the corrugation barriers. In contrast, along σ = 90°, the grains are ordered and appear more closer together with an interparticle distance *S*_2_ due to the enhanced surface diffusivity along σ = 90° (corrugation’s long axis) similar to a flat sample. The interparticle distances *S* were measured from the autocorrelation patterns, as exemplified for sample Sa2 in Figure S3. Similarly, Figure 2e and g presents the autocorrelation patterns derived from AFM topography images of samples Sa1 (flat) and Sa5 (with feature *P* ≫ *d*), respectively. Similar interparticle distances *S* along both σ = 0 and 90° were measured for samples Sa1 and Sa5, leading to lower values of Δλ_p_ as in Figure 2i and 2k as compared to sample 2 that exhibited larger value of Δλ_p_ (plasmonic anisotropy) in Figure 2j. SEM image of samples Sa1, Sa2, and Sa5 (in Figure S4) further confirms these nanometric interparticle gaps. The reflectance spectra of sample Sa1 (flat) in Figure 2i were dominated by Fabry–Perot-like interference fringes, attributed to the presence of a very thin, nonpercolated Au layer on a transparent substrate.^47^ In contrast, transmission analysis of sample Sa1 shown in Figure S5a reveals its isotropic plasmonic response. The anisotropic in-plane ordering of the grains, which determines the resulting plasmonic anisotropy, was investigated for a similar set of samples.

In these studies, the deposition conditions were optimized to promote the nucleation of Au disks with a larger diameter of *d*∼60 nm. Samples Sa4, Sa6, and Sa9 were fabricated by depositing Au films of *t* ∼ 7.5 nm onto the templates with varying *P* at a higher deposition rate of 79 Å/h. The increase in flux of particles arriving at the surface influenced the nucleation density, resulting in larger disk diameters. Figure S6a,b shows the samples with a nucleated Au disk diameter of *d* ∼ 60 nm. The Au disks in sample Sa4 with *P* ∼ 70 nm (*P* ≅ *d*) were aligned as a chain-like arrays along σ = 90°, whereas their orientation became randomized for sample Sa9 (*P* ∼ 300 nm, *P* ≫ *d*). The polarized reflectance analysis further confirmed the relationship between in-plane grain ordering and the observed plasmonic anisotropy (in Figure S6c,d), where sample Sa4 (*P* ≅ *d*) exhibits a Δλ_p_ of approximately 75 nm, whereas sample Sa9 (*P* ≫ *d*) shows a smaller Δλ_p_ value that is around 30 nm. Several works^48,49^ have reported this effect as a result of the plasmonic coupling between subwavelength MNPs under a linearly polarized excitation. The principal reason for the large anisotropy values of Δλ_p_ for samples with *P* ≅ *d* is the different interparticle distance *S* along σ = 0 and 90°, which causes different plasmon couplings and dipole decay rates between the Au disks. Furthermore, Figure S6c shows a more intense excited plasmon scattering compared to the spectra presented in Figure 2i,j,k,l, which is attributed to the stronger scattering in the case of the larger Au disk diameter (*d* ∼ 60 nm), as predicted by Mie theory.^43^ The shape of the in-plane Au disks did not appear to influence the plasmonic anisotropy, as evidenced by the lack of significant Δλ_p_ values, for both the sample with *P* ≫ *d* (Sa5) and the flat sample (Sa1) as in Figures S5a,d. They further confirm that the different coupling rates between the disks based on *S*_1_ and *S*_2_ along σ = 0 and 90° was the primary cause of the observed anisotropy. Similarly, corrugated sample Sa3 placed at the edge of the e-beam evaporator stage and coated with Au *t* ∼ 7 nm exhibited elliptically shaped Au disk nucleation on the corrugated facets. This shape results from slightly off-normal deposition occurring along the direction of *k*_*x*_ on samples placed at the edge of the deposition stage, as a result of the e-beam evaporation cone.^50,51^ Figure 2d,h shows the AFM images and their corresponding autocorrelation function patterns of sample Sa3 comprising elliptical disks. This sample with an in-plane shape aspect ratio of about 1.6:1.25 (32 nm along σ = 90° and 25 nm along σ = 0°), deposited on the template with *P* ≅ *d*, exhibited a slightly higher value of Δλ_p_ (in Figure 2l), as compared to the sample with spherical disks (Figure 2j). This is due to the combination of both shape anisotropy and interparticle coupling effects.

In order to understand the anisotropic response of the Au disk chain-like arrays, EM simulations were performed on a corrugated Al_2_O_3_/Au disk structure modeled using the optical constants for Au described by Yakubovsky et al.^52^ for a 6-nm-thick Au film. Figure S7a,b shows the simulated polarized reflectance spectra obtained for the angles σ = 0, 45, and 90°, for samples Sa2 and Sa3 with spherical and elliptical Au disks, respectively. These simulations are presented in Figure S7a and show similar anisotropic trends at σ = 0 and 90° to those observed experimentally in Figure 2j but with slightly red-shifted λ_TMp_ and λ_TEp_ resonance positions at σ = 0 and 90°. This red shift can be attributed to imperfections in the arrangement of Au disk ordering on the fabricated sample surface. In contrast, the perfectly periodic Au disk arrays modeled in simulations exhibit stronger plasmon coupling, resulting in the red shift of λ_TMp_ and λ_TEp_ resonance positions to longer wavelengths due to plasmon hybridization.

### Plasmonic Hybridization in 2D-MNP Arrays

“Collective plasmonic modes” arising from the interaction of near-fields in ordered MNP chains with *S > r* as observed in this study, as well as its resulting plasmonic anisotropy dependent on σ, can be described by plasmon hybridization theory.^16,53,54^ Hybridization of plasmonic modes can significantly enhance light absorption (as dark modes) and scattering (as bright modes). However, dark modes cannot be directly excited by linearly polarized free space light due to their vanishing oscillator strength in the symmetric MNP chain. In contrast, bright modes, with a net oscillator strength due to their dipoles being aligned in-phase, can be excited by free space linearly polarized light.^53,55^ It is the hybridized bright modes that manifest themselves in the λ_p_ positions along TE (σ = 90°) and TM (σ = 0°) polarizations as longitudinal (bonding) and transverse (antibonding) dipole modes on dimers and 1D-MNP chains. As a result of this hybridization, depending on the interparticle distance *S* and the number of particles *N*, the anisotropic plasmonic behavior arises in dimers and 1D-MNP chains.^16,56^ In the fabricated 2D-MNP chains, both polarizations along σ = 90° and σ = 0° can be considered to excite hybridized bonding plasmon modes as λ_TEp_ and λ_TMp_, respectively; see Figure S7a. Here, the anisotropy arises from the excitation of higher-order modes, based on the different interparticle distances *S*_1_ and *S*_2_ along σ = 0 and 90°, respectively. Excitation of both higher-order dark modes at λ_p2,4_ (characterized by even integer fractions 2 and 4 of the fundamental dipole mode wavelength) and higher-order bright modes at λ_p3,5_ (characterized by odd integer fractions 3 and 5 of the fundamental dipole wavelength) cannot be neglected for systems with very small interparticle gaps as *S ≤ r*.^16,56^ These higher-order modes push the lower-order dipole mode to longer wavelengths, causing the red-shifting and broadening that are observed in the ordered MNPs with *S ≤ r*. While dark modes are not observable in optical far-field spectroscopy, bright higher-order modes are visible only for MNP chains with finite *N* (typically *N* ≤ 5). However, in this study, where *N* is assumed to be very large, the higher-order bright modes further red shift and then degenerate into the peak corresponding to the lowest-order dipole mode λ_p_ ^16,53,56^ as λ_TMp_ at σ = 0° and λ_TEp_ at σ = 90° based on the interparticle gap *S* at these angles. Further, these higher-order bright modes are designated to be subradiant in their nature as the induced surface charge density at λ_p3,5_ forms dipole-like characteristics in the individual MNPs that alternates along the MNP chain reducing its overall dipole moment collectively.^16,53^ As a result, these bright subradiant λ_p3,5_ modes contribute to less radiative scattering when compared to the super-radiant λ_p_ (dipole antenna mode) that is also a collective plasmon in the arrays but effectively radiates the coupled field into far-field by dynamic depolarization due to its nonzero dipole moment collectively. This change in the effective modal index of these particle chains at λ_p3,5_, which supports propagating subradiant SPP modes, can be advantageous for increasing the plasmon lifetime. Attributed to both efficient light collection into the near-field and reduced far-field radiation due to the plasmon propagation, these subradiant modes excited at the wavelengths λ_p3,5_ < (λ_p_ lower-order dipole mode) due to hybridization, occurs only when *S* ≪ *r* for ordered MNP chain arrays as in our experimentally studied samples. Further, this phenomenon becomes evident when the far-field optical spectra are deconvoluted into scattering and absorption components that separate the contributions of far-field radiative losses from efficient light coupling.^57,58^ Several studies^16,53,54^ have also reported the effect of the excitation of the hybridized-collective SPP mode in ordered systems with *S ≤ r*. This opens a route for advancements in a wide range of applications such as wave-guiding and on-chip communication.^59–61^ Furthermore, simulations were performed to analyze the near-field spatial distribution and corroborate it with polarized far-field reflectance and transmission measurements to characterize these hybridized-collective plasmon modes. Figure 3a and 3b shows the normalized 2D near-field distribution for sample Sa2 with spherical Au disks excited at its resonance peak maximum positions of λ_exc_ = 639 nm at σ = 0° and λ_exc_ = 715 nm at σ = 90°. The near-field distribution clearly show that the restoring force is reduced because *S* ≪ *r* at both σ = 0 and 90° in these 2D-MNP arrays.^62,63^ They confirm the excitation of the hybridized-collective plasmon modes with giant near-field enhancements, which accounts for the red shift of both the plasmon resonances at σ = 0 and 90° beyond the uncoupled single-particle λ_p_ of sample Sa11 with deposited Au *t* ∼ 2 nm (low metal fill-factor) in Figure S2b. Further, we also present the simulated near-field distribution of sample Sa2 obtained at different excitation wavelengths of 600, 639, 675, 715, and 785 nm in Figure S8 to compare and correlate the excitation positions of experimental SERS measurements and the simulations with respect to their plasmon resonance peak positions.

**Figure 3:**
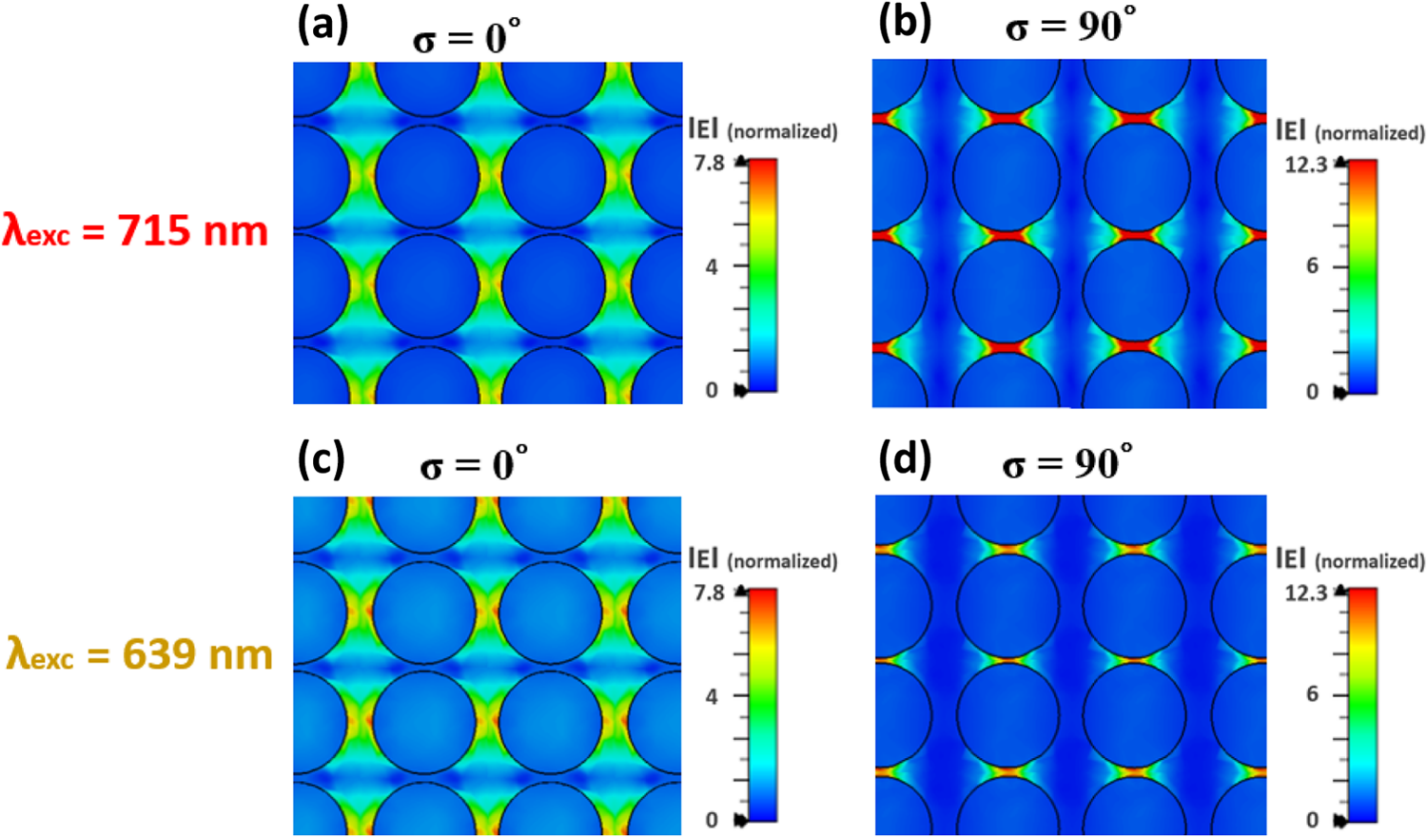
(a, b) Simulated EM near-field distribution for sample Sa2 at λ_exc_ = 715 nm for σ = 0 and 90°, respectively. (c, d) EM near-field distribution images for sample Sa2 at λ_exc_ = 639 nm for σ = 0 and 90°, respectively. The selected field distributions are presented for (3 × 3) cell fragments of infinite periodic arrays with the Au disk/Al_2_O_3_ structural properties of sample Sa2 and were performed at the red-shifted resonance maximum positions to investigate and distinguish the near-field intensity and more importantly their spatial distribution at both polarization angles (σ = 0 and 90°).

These results also reveal that the strongest coupling occurs at σ = 90° for both wavelengths, regardless of the λ_TMp_ peak position at 639 nm for σ = 0° and the λ_TEp_ peak position at 715 nm for σ = 90° in the simulated spectra. This is attributed to the shorter interparticle distance *S*_2_ along σ = 90°. However, there is a moderately increased spatial distribution at λ_exc_ = 715 nm, where the λ_TEp_ resonance at σ = 90° is at its maximum. However, for σ = 0° due to the larger value of *S*_1_ along this polarization angle, the near-field was not as strong as for σ = 90° at both excitation wavelengths even for λ_exc_ = 639 nm where the λ_TMp_ for σ = 0° is at its maximum. Due to this generation of giant near-field enhancements with anisotropic response, polarized and wavelength-scanned SERS measurements were performed for thiophenol molecules in these samples, in order to investigate the SERS due to these hybridized-collective plasmon modes and study SERS enhancement mechanism by analyzing its relative Raman peak intensities.

### Polarized and Wavelength-Scanned SERS Measurements

Apart from simulated near-field distributions discussed in the previous section, the enhanced near-field *E*_p′_(λ_p′_) related to these hybridized-collective plasmon modes can be measured in SERS experiments, as plasmonic modes with enhanced fields *E*_p_(λ_p_) can influence different stages of Raman enhancement of a molecule depending on the enhancement factors *g* at λ_exc_ and *g* ^*s*^ at λ_Stokes_ (see eq 3). The conducted experimental studies utilizing plasmonic anisotropy allow us to retrieve the individual influence of *g* and *g* ^*s*^ on the SERS signals of the investigated analyte. The fabricated samples were dipped in solution of organic thiophenol molecules to form a layer of phenyl thiolates onto the Au surface and then dried for a few minutes. Two selected Raman excitation laser wavelengths λ_exc_ at 633 and 785 nm related to the sub- and super-radiant plasmon modes were used in the study. The vertical lines in Figures 4a,b and S5 indicate the incident λ_exc_ and its corresponding λ_Stokes_ positions on the plotted polarized reflectance and transmission spectra of samples Sa1 (unordered grains: flat sample), Sa2 (ordered spherical arrays: *P* ≅ *d*), Sa5 (unordered spherical arrays: *P* ≫ *d*), and Sa3 (ordered elliptical arrays: *P* ≅ *d*). Since SERS measurements are performed using a microscopic objective with a high numerical aperture 0.7 *N*.*A*., focusing within an aperture angle of 50°, we prefer to align the λ_exc_ and λ_Stokes_ positions on the reflectance spectra measured at θ = 50° (see Figure 4a,b), rather than with the transmission spectra that were obtained at normal incidence (see Figure S5). Shown in Figure 2i, transmission spectra were measured to analyze the Δλ_p_ of the flat sample (Sa1) as its reflectance analysis in Figure 2i exhibited interference fringes rather than the λ_p_ position due to the very small value of *t* on transparent-sapphire substrate. Importantly, off-normal reflectance analysis at θ = 50° is necessary to account for the out-of-plane (*p*-polarized) light interaction with the plasmonic surface, which can excite both parallel and perpendicular charge oscillations.^64^

**Figure 4:**
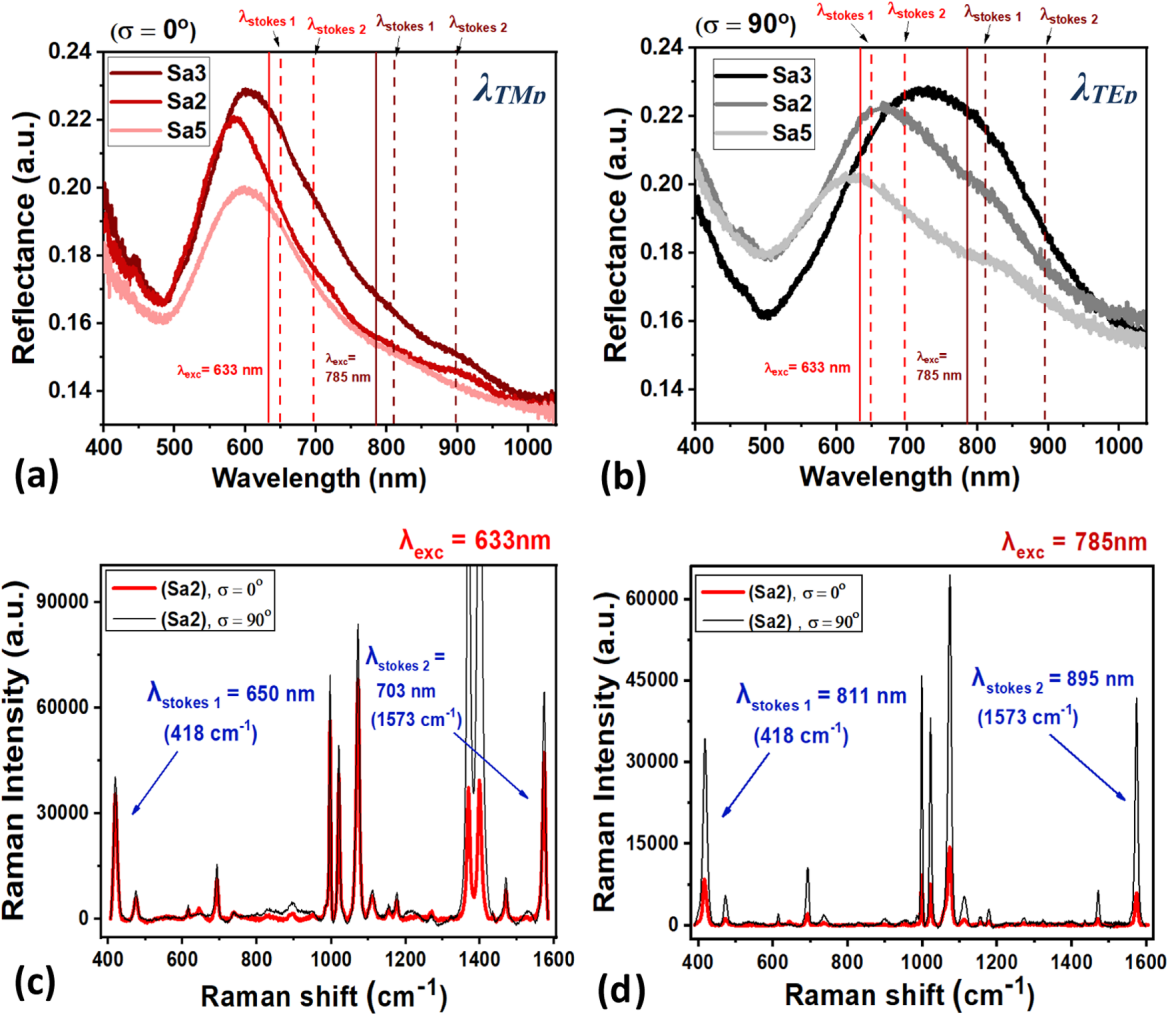
Polarized reflectance spectra (a) at σ = 0° (red lines) exhibiting the excited λ_TMp_ plasmon modes and (b) at σ = 90° (black lines) exhibiting the λ_TEp_ plasmon modes for samples Sa2, Sa3, and Sa5 (measured at an angle of incidence *θ =* 50°). The vertical lines indicate λ_exc_ and its corresponding λ_Stokes_ positions on the spectra. (c) SERS spectra measured at λ_exc_ = 633 nm for sample Sa2 at both σ = 0 and 90° polarizations. (d) SERS spectra measured at λ_exc_ *=* 785 nm for sample Sa2 at both σ = 0 and 90° polarizations. Blue arrows indicate the λ_Stokes *1*_ and λ_Stokes *2*_ positions in panels (c) and (d).

This phenomenon is important when studying optical properties of metallic thin-films and arrays on dielectric substrates with high permittivity (*ε* = 11 as for sapphire*)*. In such samples, the discontinuity of *ε* due to the underlying template breaks the symmetry of the dielectric medium surrounding the Au grains introducing phenomena such as image charges;^65,66^ these image charges interact with the excited plasmon mode and further increase the FWHM of its λ_p_ peak. Such dielectric substrates with high permittivity efficiently concentrate the *EM* near-field at the metal/dielectric interface as confirmed by recent experimental studies.^67^ Further analysis of results presented in Figures 4a, 5b, and S5 reveals that the positions of λ_exc_ = 785 nm and its corresponding λ_Stokes_ lie in the lower-frequency region compared to the peak maxima of the lower-order dipole modes of λ_TEp_ at σ = 0° and λ_TMp_ at σ = 90° for samples Sa1, Sa2, Sa3, and Sa5. However, λ_exc_ = 633 nm and its corresponding λ_Stokes_ lies in the higher-frequency region of λ_TEp_ at σ = 90° for samples Sa2 and Sa3, where the degenerated λ_TEp3,5_ subradiant propagative (SPP mode) is supported.^16,53^ Figure 4c and 4d shows the SERS spectra measured at both λ_exc_ = 633 and 785 nm along σ = 0 and 90° for sample Sa2. It is demonstrated that higher SERS intensities are acquired at λ_exc_ = 633 nm near the frequency of the subradiant propagative SPP mode with longer lifetime, when compared to λ_exc_ = 785 nm region that is related to the lower-order dipole antenna mode. However, the anisotropy in SERS intensity between σ = 0 and 90° was more prominent for λ_exc_ = 785 nm than at λ_exc_ = 633 nm. These samples exhibited SERS enhancement factors up to 10^6^ orders (see calculations in the Supporting Information). In order to acquire more detailed dependence, polarized SERS measurements with a step of Δ*σ* = 5 ° were performed on these samples. Figure 5a–d shows the polarized SERS intensity plotted for selected phenyl thiolates Stokes-shifted peaks at 418 cm^–1^ and 1573 *cm* ^–1^ designated as λ_Stokes 1_ and λ_Stokes 2_ based on their respective Raman emission wavelengths. The λ_Stokes 1_ peak is close to λ_exc_ and can be expected to exhibit an enhancement factor of *g* ≅ *g* ^*s*^. The SERS intensity of this peak can be approximated through the commonly used *|E|* ^4^ mechanism.^68,69^ However, for the higher-frequency Stokes-shifted peaks, such as the one at 1573 cm^–1^ (λ_Stokes 2_), both the factors *g* at λ_exc_ and *g* ^*s*^ at λ_Stokes_ will differ and influence the SERS intensity separately based on the position and FWHM of the excited plasmon mode. The polarized SERS peak intensities at both λ_exc_ = 633 nm (left column) and λ_exc_ = 785 nm (right column) in Figure 5a–d provide a clearer depiction of the dichroic SERS effect in these samples and allow one to directly compare their intensities and anisotropies. The flat sample Sa1 (black plots) exhibits an isotropic SERS scattering effect for both excitation wavelengths (in Figure 5). Further, in Figure 5a and 5c for λ_exc_ = 633 nm < λ_TEp_, the absolute SERS intensity values at σ = 90° angle are similar between samples Sa1 (unordered grains on the flat template: black dots), Sa2 (ordered spherical chain array along σ = 90°: red dots), and Sa5 (unordered spherical chain array along σ = 90°: green dots).

**Figure 5:**
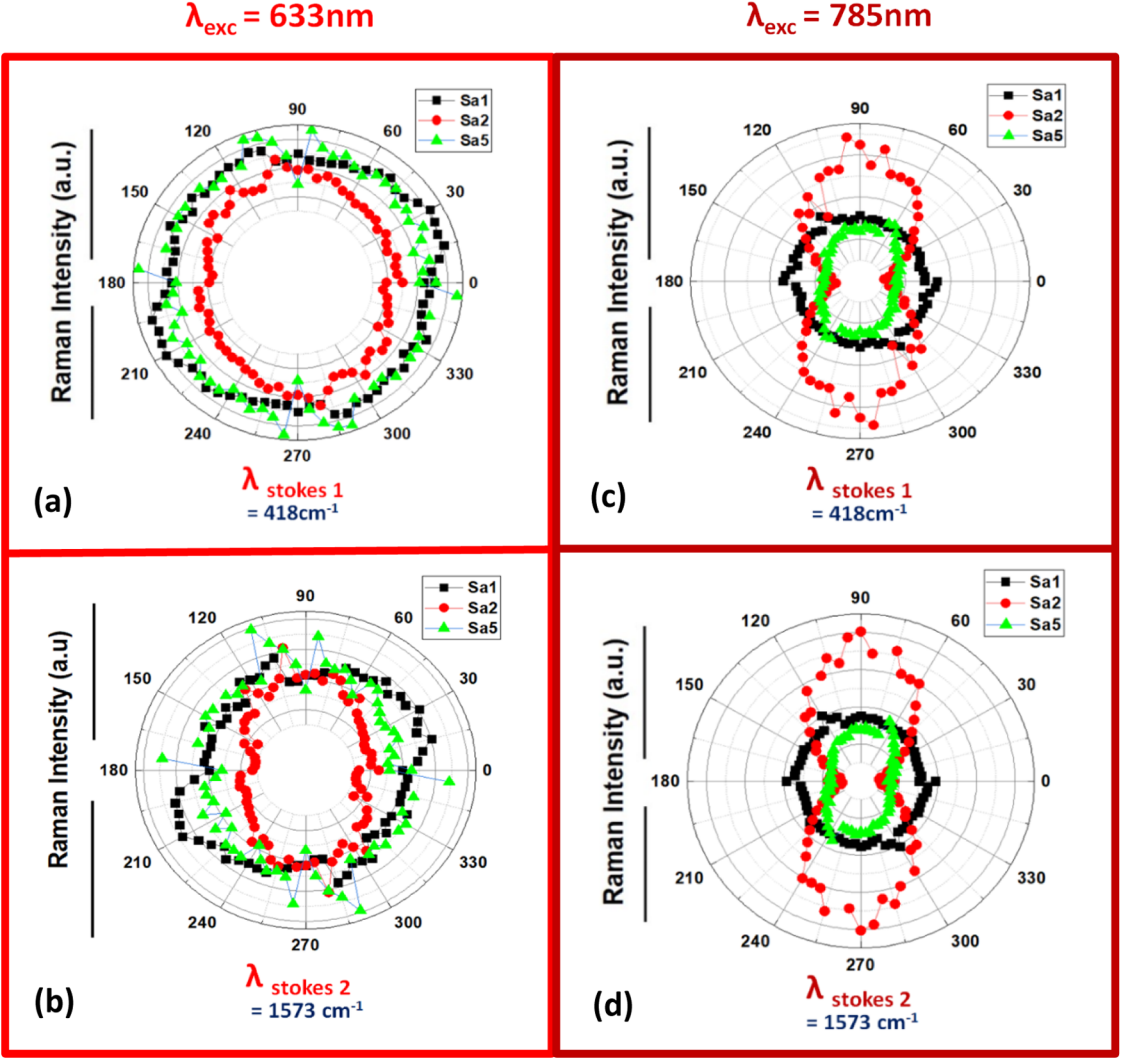
(a, c) SERS intensity dichroism plots of λ_Stokes 1_ and λ_Stokes 2_ peaks on samples Sa1 (unordered grains on the flat template: black dots), Sa2 (ordered spherical arrays: red dots), and Sa5 (unordered spherical arrays: green dots) measured for Δ*σ* = 5 ° at λ_exc_ = 633 nm. (b, d) SERS intensity dichroism plots of λ_Stokes 1_ and λ_Stokes 2_ peaks on samples Sa1 (unordered grains on the flat template: black dots), Sa2 (ordered spherical arrays: red dots), and Sa5 (unordered spherical arrays: green dots) measured for Δ*σ* = 5° at λ_exc_ = 785 nm.

This reveals that at λ_exc_ = 633 nm, where λ_exc_ < λ_TEp_, near the frequency of the subradiant collective plasmon mode, its resulting SERS enhancement depends primarily on the interparticle gap spacing *S*, rather than the ordering of MNP as arrays when *S* ≪ *r*. These similar values of absolute SERS intensities between Sa1, Sa2, and Sa5 along σ = 90° contradict the far-field reflectance intensities of Sa2 and Sa5 in Figure 5b that show higher reflectance for Sa2 when compared to Sa5 at λ_exc_ = 633 nm and its corresponding λ_Stokes_ positions (compare Sa2 and Sa5 intensities at light red lines position in Figure 5b). This anomalous dependency of SERS at λ_exc_ = 633 nm on the far-field reflectance is due to the increase in the spatial and temporal (lifetime) features of the enhanced near-field due to plasmon propagation with reduced far-field scattering, as these subradiant modes are excited due to hybridization in arrays with *S* ≪ *r* and cannot interact (couple or out-couple) with far-field radiation. Due to this dependence on *S*, at σ = 0° polarization, only sample Sa2 with a large interparticle gap *S*_1_ showed a slight decrease in SERS intensity causing a dichroic response (see the red plot in Figure 5a and 5c). This effect is specific to sample Sa2 (*P* ≅ *d*) at σ = 0°, where the corrugation barrier causes an increase in the value of *S*_1_. Similarly, for the flat sample Sa1 and sample Sa5 (*P* ≫ *d*), the interparticle gap *S* is similar at both σ = 0 and 90° angles, resulting in an isotropic SERS effect under λ_exc_ = 633 nm; see Sa1 (black plot) and Sa5 (green plot) in Figure 5a and 5c.

In contrast, when analyzing the data for λ_exc_ = 785 nm in Figure 5b and 5d, there is a clear difference in SERS absolute intensities between the flat Sa1, Sa2, and Sa5 samples at σ = 90°. This observation is in agreement with the changes in far-field polarized reflectance intensities between those samples at σ = 90° for λ_exc_ = 785 nm and its respective λ_Stokes_ positions (compare Sa2 and Sa5 intensities at dark red lines position in Figure 4b). At Raman λ_exc_ = 785 nm > λ_TEp_ related to the frequency of the lowest-order dipole mode, the ordering of *MNPs* in the form of arrays for sample Sa2 (red plot) in Figure 5b and 5d primarily influenced the increase in its SERS signal and corroborates well with its far-field reflectance spectra. This is due to the excited lowest-order dipole mode (antenna mode) that is effectively excited in the array as collective plasmons but instantly scatters the radiation into far-field as a result of dynamic depolarization; this is due to the nonzero dipole moment in ordered arrays collectively at λ_exc_ = 785 nm > λ_TEp_. This is the reason why the absolute SERS intensities at λ_exc_ = 785 nm at σ = 90° decreased for the unordered array samples Sa1 and Sa5 along σ = 90° (see Figure 5b and 5d) when compared to (Sa2) with the ordered spherical chain array along σ = 90° even though all of the samples possessed the same interparticle gap *S*_2_. Furthermore, sample Sa2 exhibited a pronounced SERS dichroic effect (see Figure 5b and 5d) dependent on far-field polarization angle (σ) due to the effective coupling of the dipole antenna mode at σ = 90° on the ordered arrays as discussed. Subsequently, the dichroism becomes less pronounced for the sample with nonordered arrays (Sa5), and it completely disappeared for the flat sample (Sa1). Similarly, when comparing the absolute SERS intensities between these samples at σ = 0° under λ_exc_ = 785 nm, the flat sample Sa1 with the smallest *S* value exhibited the highest SERS intensity and sample Sa2, with the largest gap distance *S*_1_ at σ = 0°, exhibited the lowest SERS intensity (see data at σ = 0° in Figure 5b and 5b).

The most enhanced SERS intensities in these samples with subwavelength Au disk dimension and periodicity were obtained for incident laser wavelength λ_exc_ = 633 nm (λ_exc_ < λ_TEp_) near the frequency of the subradiant collective plasmon mode (Figure 4c) due to its increased spatial and temporal features, when compared to spectra collected for λ_exc_ = 785 nm (λ_exc_ > λ_TEp_) related to the frequency of the lowest-order (antenna) dipole mode (Figure 4d). These experimental observations comparing the SERS intensities and anisotropies of the samples, excited at different λ_exc_ and polarization angles (σ), summarize that in the SERS system with *S* ≪ *r*, the interparticle gap distance *S* is the primary factor influencing SERS enhancements compared to the factor of ordering MNPs in the form of arrays. This dependence becomes significant when designing large-area SERS substrates based on arrays with very small interparticle gaps since they exhibit larger number of intense SERS hotspots in real space.

Furthermore, when analyzing the polarized SERS peak intensities of sample Sa3 (*P* ≅ *d*, ordered elliptical arrays), the relative SERS peak intensities between λ_Stokes 1_ and λ_Stokes 2_ were distinct at σ = 0 and 90°, as shown in Figure 6a. At σ = 0° (red spectra), the λ_Stokes 1_ peak is enhanced strongly as compared to the λ_Stokes 2_ peak, while the reverse is observed at σ = 90° (black spectra). This effect of change in the relative SERS intensities dependent on σ is only visible at λ_exc_ = 633 nm, while at λ_exc_ = 785 nm, the SERS anisotropy behaves similar to that observed for samples Sa2 and Sa5 (Figure 4b and d). When the polarized SERS intensities of λ_Stokes 1_ and λ_Stokes 2_ peaks at λ_exc_= 633 nm were plotted with a step Δ*σ* = 20° (Figure 6b), the analysis revealed distinct dichroic response between λ_Stokes 1_ and λ_Stokes 2_ peaks. Specifically, λ_Stokes 1_ exhibits a cos^2^(σ) dependency (black dots), while λ_Stokes 2_ exhibits a sin^2^(σ) dependency (red dots) with respect to the polarization angle σ. By corroborating these results with the λ_exc_ = 633 nm and its corresponding λ_Stokes_ in the polarized reflectance and transmission plots (see Figures 4a, 5b, and S5c), it was observed that both λ_exc_ = 633 nm and its corresponding λ_Stokes 1_ are closer to the λ_TMp_ position at σ = 0°. This results from the fact that SERS peak intensity related with the λ_Stokes 1_ depends on the enhancement factors assuming that *g* ≅ *g* ^*s*^. In contrast, for higher-frequency Stokes-shifted peak λ_Stokes 2_ due to the differing values of *g* and *g* ^*s*^ at σ = 0°, a reduced SERS peak intensity is observed at σ = 0° as the λ_TMp_ resonance is blue-shifted with respect to λ_Stokes 2_, and an increase in its peak intensity was observed at σ = 90° where the field *E*_p_ at λ_TEp_ is enhanced at the λ_Stokes 2_ position. This confirms that the intensities of λ_Stokes_ depend on both enhancement factors *g* and *g* ^*s*^, which are determined by the λ_exc_ and its corresponding λ_Stokes_ positions relative to the excited plasmon mode. These experimental results emphasize the requirement of plasmon resonance with broad resonance peaks for the enhancement of both *g* and *g* ^*s*^ efficiently in SERS, where the fabricated arrays with interparticle gap *S ≤ r* are preferred. Similar experimental results were also obtained for other samples presented in Figures S9, S10, and S11. These findings further validate the EM near-field enhancement mechanism of SERS, based on these SERS responses due to the anisotropic near-field enhancements. The fabricated samples exhibited capability of highly distributed hotspot (2 × 2 cm arrays), SERS enhancement of 10^6^ orders, and broad peaks of the collective plasmon modes for the enhancement of both *g* and *g* ^*s*^ efficiently. SERS mapping measurements performed in different polarization angles and excitation wavelengths in Figures S12, S13, S14, and S15 exhibit long-range uniform sensitivity of the fabricated samples. Finally, as a suggestion for the continuation of this study, transient absorption spectroscopy can be used to experimentally investigate the lifetime of these hybridized-collective plasmon modes in a quantitative way and corroborate it with SERS enhancements resulting from incident excitation wavelength λ_exc_ at subradiant frequency regimes.

**Figure 6:**
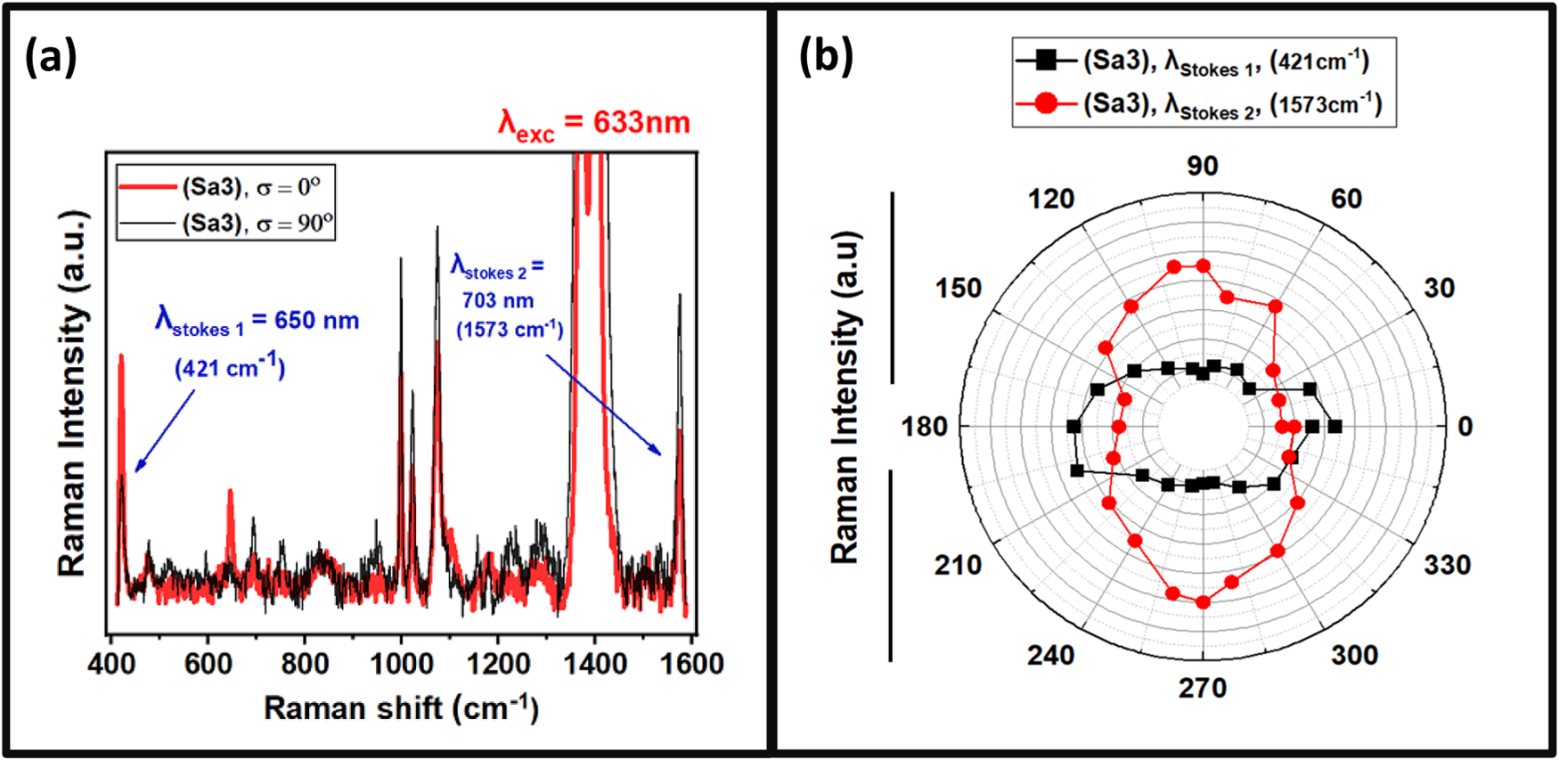
(a) SERS spectra measured at λ_exc_ = 633 nm for sample Sa3 for both σ = 0 and 90° polarizations. (b) Polarized SERS intensities of λ_Stokes 1_ and λ_Stokes 2_ peaks at λ_exc_ = 633 nm for sample Sa3, revealing that λ_Stokes 1_ follows a cos^2^(σ) and λ_Stokes 2_ follows a sin^2^(σ) dependency.

## Conclusions

Ordered 2D-MNP arrays with interparticle gap *S* ≪ *r*, where *r* is the radius of the MNP, were fabricated by depositing nonpercolated Au films onto corrugated sapphire templates. The optimal condition for the ordering of MNPs as arrays was achieved at a low Au deposition rate of 10 Å/h when utilizing an e-beam evaporation method under ultrahigh vacuum (10^–10^ mbar). The Au grains nucleated with anisotropic in-plane ordering onto templates with feature *P* ≅ *d*, where *d* is the diameter of the grain. Polarized reflectance analysis exhibited an anisotropic plasmonic response in the ordered Au arrays dependent on the angle between the corrugation direction and laser polarization direction (σ). Furthermore, simulated spectra and EM near-field distributions corroborated the anisotropic plasmonic trend and revealed the excitation of hybridized (bonding) collective plasmon modes as λ_TMp_ along σ = 0° and λ_TEp_ along σ = 90°. This hybridization excited higher-order plasmon modes due to the strong coupling between the MNPs with nanometric interparticle gap. Further, SERS enhancements of phenyl-thiolate analytes excited at λ_exc_ = 633 nm (λ_exc_ < λ_TEp_) (near the frequency of the subradiant collective plasmon mode) depended primarily on the interparticle spacing *S* rather than the ordering of MNPs in the form of arrays. This conclusion is based on the results of similar absolute SERS intensities observed for λ_exc_ = 633 nm at σ = 90° for samples Sa1 (unordered grains: flat sample), Sa2 (ordered spherical chain arrays along), and Sa5 (unordered spherical chain arrays along). On the contrary, at λ_exc_ = 785 nm, the absolute SERS intensities differed between samples Sa1, Sa2, and Sa5 along σ = 90°. This reveals that under λ_exc_ = 785 nm (λ_exc_ > λ_TEp_) related to the collective dipole plasmon mode, the ordering of MNPs as arrays amplified the antenna modes far-field interaction, subsequently resulting in a highly anisotropic SERS enhancement dependent on far-field polarization σ. We demonstrated, through experiment comparing the SERS intensities and anisotropies of these samples excited at different λ_exc_ and polarization angle σ, that in systems with *S* ≪ *r*, the interparticle gap size *S* was the primary influencing factor toward SERS enhancements when compared to factor of ordering MNPs in the form of arrays. Furthermore, the plasmonic anisotropy-induced SERS dichroism effect depended on the position of λ_exc_, its corresponding λ_Stokes_ with respect to the excited plasmon mode. As a result of this, the lower-frequency Stokes-shifted peak at λ_Stokes 1_ exhibited a cos^2^(σ) dependency following the *|E|* ^4^ model. At the same time, the higher Stokes-shifted peak λ_Stokes 2_ exhibited a sin^2^(σ) dependency as its SERS intensity depended on both the distinct near-fields *E*_exc_ enhanced at λ_exc_ and *E*_Stokes_ enhanced at λ_Stokes_, verifying the EM near-field mechanism of SERS. Seminal requirements such as distributed hotspot (2 × 2 cm arrays), broad resonance peak (enabling wider Stokes-shifted ranges), and SERS enhancements up to 10^6^ orders were achieved in order to enable reliable, efficient, and robust SERS sensing.

## Supplementary Material



## Notes

Additional information and schematics about the experimental and structural parameters of the fabricated samples; additional AFM and SEM images of the studied sample; polarized reflectance and transmission measurements of the studied sample; schematics explaining the meaning of interparticle gap spacing *S;* simulated far-field optical spectra and EM near-field distributions, SERS mapping, polarized and wavelength-scanned micro-Raman spectra of the studied samples; and calculations of SERS enhancement factors (PDF)

## Abbreviations

1D and 2D: one-dimensional and two-dimensional
MNP: metal nanoparticle
SERS: surface-enhanced Raman scattering
EM: electromagnetic
LSPR: localized surface plasmon resonance
FWHM: full width at half-maximum
e-beam: electron beam
TE and TM: transverse electric and transverse magnetic
SPP: surface plasmon polariton
