# Supplementary material for: Raman Scattering Enhancements Due to Super- and Subradiant Collective Plasmon Modes on Large-Area 2D-Au Arrays
Source: ACS Appl Mater Interfaces. 2025 May 22;17(22):33176–90. doi: 10.1021/acsami.5c04804 (PMC12147072; doi:10.1021/acsami.5c04804)
Supplement: Supplementary file 1 [file am5c04804_si_001.pdf]

SUPPORTING INFORMATION

Raman Scattering Enhancements Due to Super and Subradiant Collective Plasmon Modes on Large-Area 2D - Au Arrays

Ephraim T. Mathew[1,2], Andriy E. Serebryannikov[1], Jacek Jenczyk[2], Igor Iatsunskyi[2], Szymon Murawka[2], Mikołaj Lewandowski[2], and Maciej Wiesner[1,]*

*Corresponding author's email: mwiesner@amu.edu.pl

[1] Faculty of Physics and Astronomy, Adam Mickiewicz University,

Uniwersytetu Poznańskiego 2, 61-614 Poznań, Poland

[2] NanoBioMedical Centre, Adam Mickiewicz University,

Wszechnicy Piastowskiej 3, 61-614 Poznań, Poland

SUPPORTING INFORMATION

Table S1. Experimental and structural parameters of the fabricated samples. Measurement of Au grain diameter *d* was not possible for percolated and near-percolating films with connected *MNP*s network for Au thickness (t >7.5 nm)[1,2].

Sample name	Sa4	Sa6	Sa9	Sa11	Sa12	Sa13	Sa14	Sa15	Sa16	Sa17
**(P) corrugation period [nm]**	70 ± 8 nm	150 ± 25 nm	240 ± 25 nm	290 ± 25 nm	290 ± 25 nm	290 ± 25 nm	290 ± 25 nm	290 ± 25 nm	290 ± 25 nm	70 ± 25 nm
**(h) Corrugation height [nm]**	8 ± 2 nm	18 ± 5 nm	25 ± 5 nm	30 ± 5 nm	30 ± 5 nm	30 ± 5 nm	30 ± 5 nm	30 ± 5 nm	30 ± 5 nm	8 ± 2 nm
**(t) Au thickness [nm]**	~8 nm	~8 nm	~8 nm	~2 nm	~4 nm	~6 nm	~7 nm	~8 nm	~10 nm	~7 nm
**(d) Au grain diameter (nm)**	60 ± 5 nm	60 ± 5 nm	60 ± 5 nm	25 ± 5 nm	25 ± 5 nm	27 ± 5 nm	27 ± 5 nm	-	-	27 ± 5 nm
**Au deposition rate (Å / hour)**	79	79	79	10	10	10	10	10	10	10

SUPPORTING INFORMATION

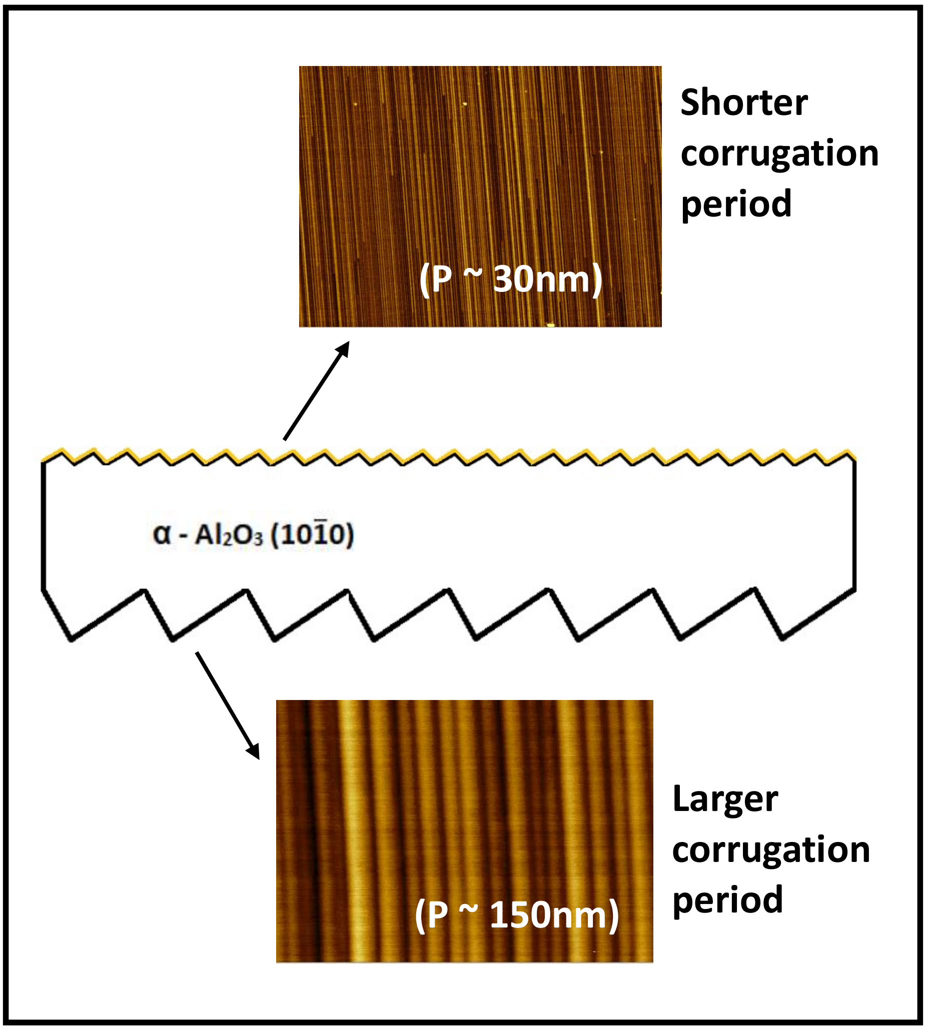

Figure S1. AFM images with schematics exhibiting formation of corrugations with different periods on both sides of the reconstructed double side polished M-plane [$10\bar{1}0$] wafer[3].

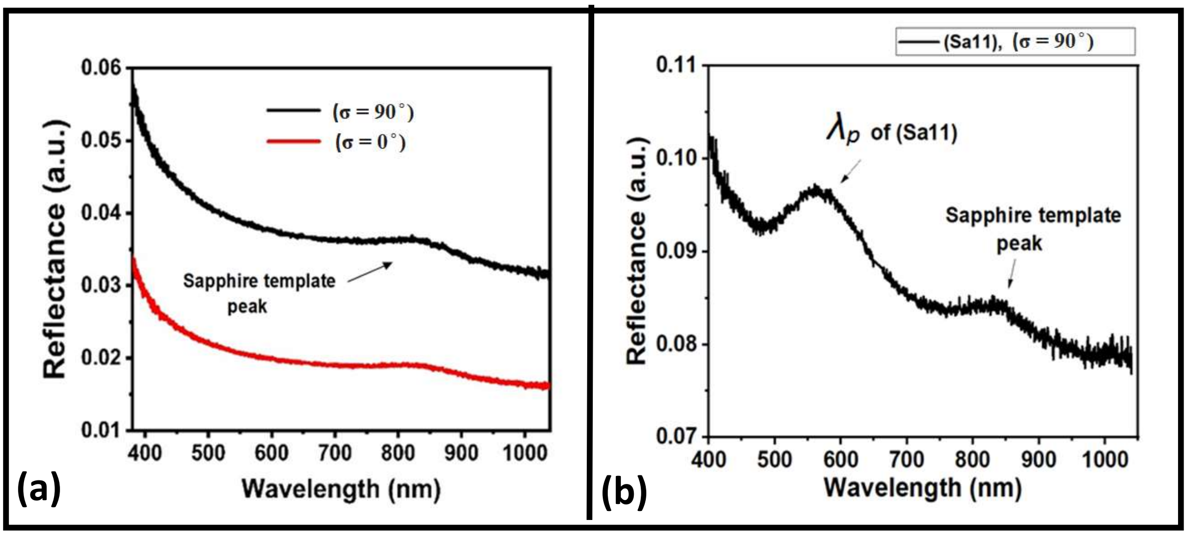

Figure S2. (**a)** Reflectance measurement on a bare, non-reconstructed M-plane sapphire template with an elevated peak at 831 nm. **(b)** Reflectance measurement of Sa11 with 4 nm thick Au showing sapphire template peak at 831 nm and λ_p of Sa11 around 575 nm.

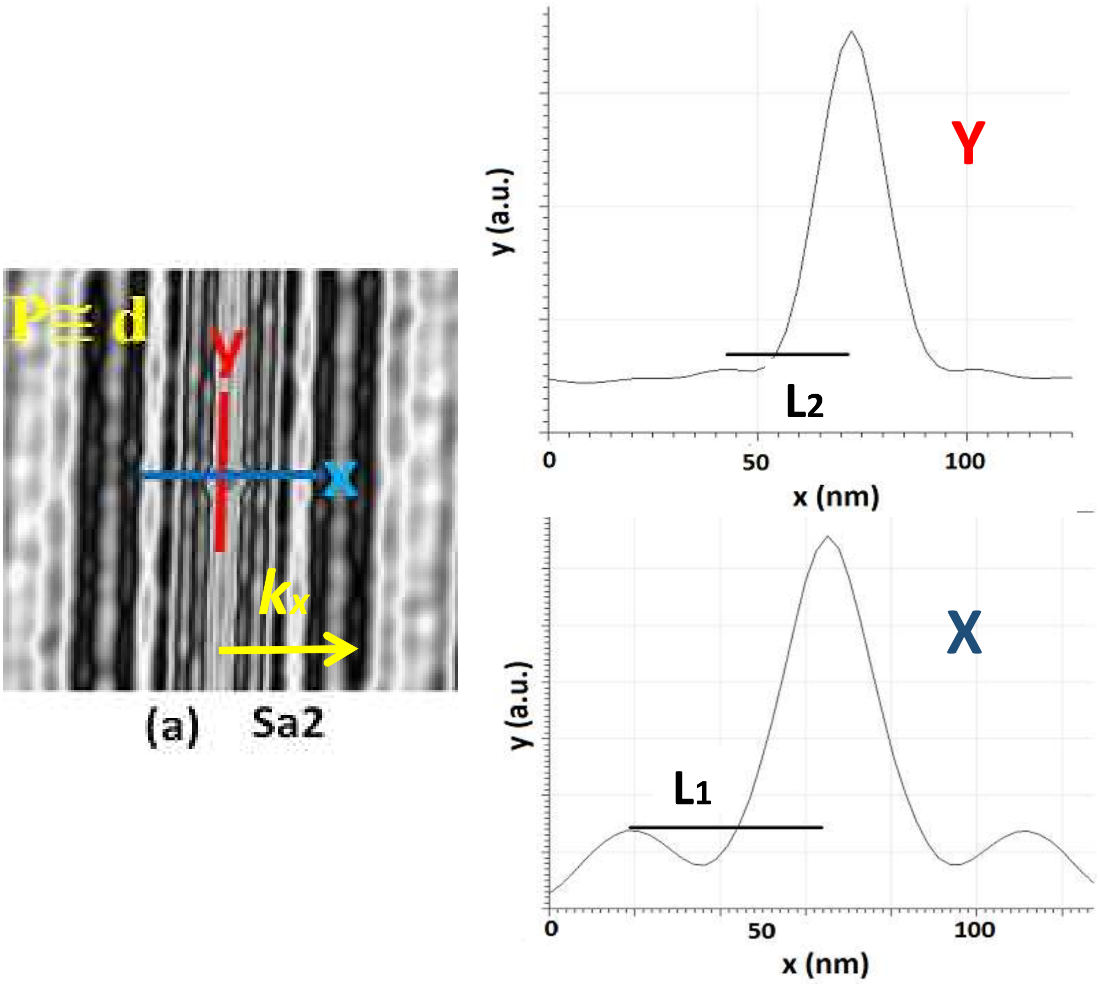
P≅d
Y
X
kx
(a) Sa2
y (a.u.)
Y
L2
0
50
100
x (nm)
y (a.u.)
X
L1
0
50
100
x (nm)

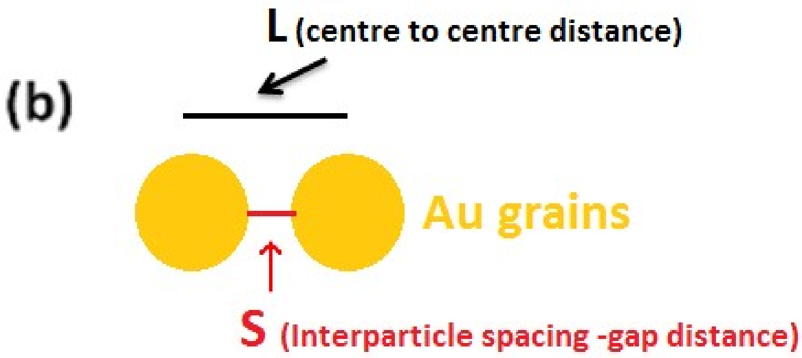
(b)
L (centre to centre distance)
Au grains
S (Interparticle spacing -gap distance)

Figure S3. (**a)** Autocorrelation functions of the sample Sa2 and its respective line graphs from where their interparticle distances S_1 and S_2 were determined. (**b)** Schematics explaining the meaning of interparticle spacing *S*. The calculated values of interparticle gap distance S_1 ~3 nm and S_2 ~ 1 nm for sample Sa2.

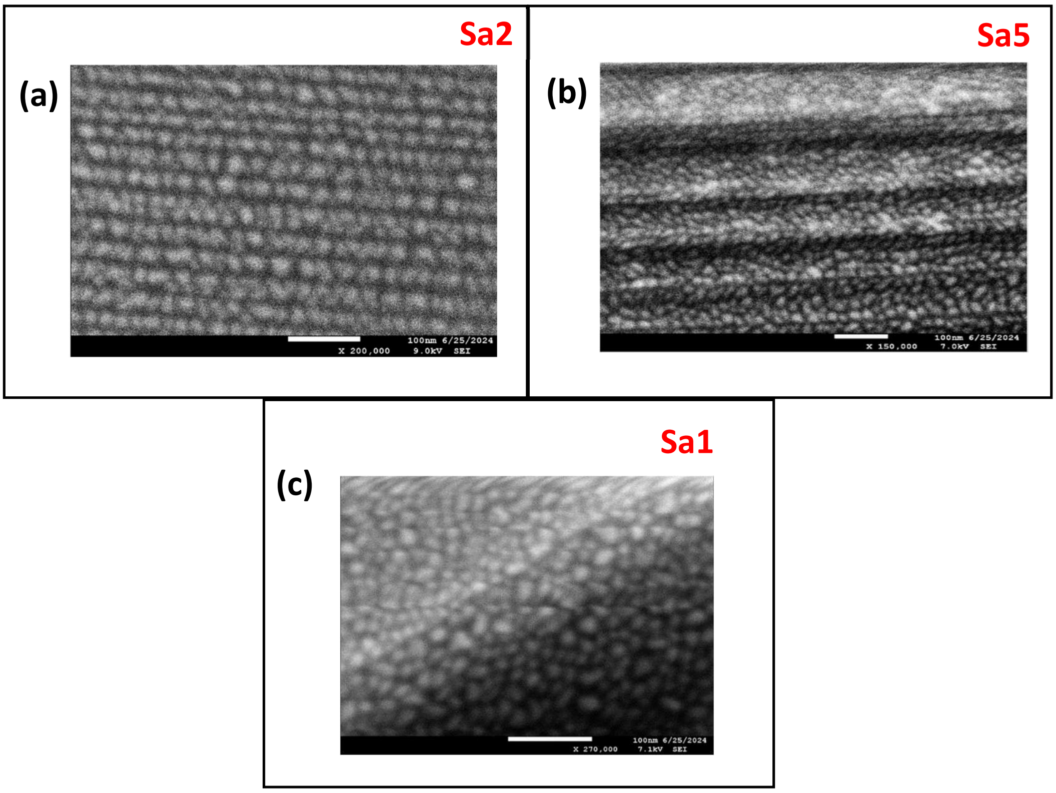

Figure S4. SEM images of sample (**a**) Sa2 with P≅ d, (**b**) Sa2 with P>> d, and (**c**) Sa1 (flat). *The SEM images were very poor*. This is because the sapphire is a highly insulating material with a large dielectric constant. Its poor electrical conductivity led to significant charging effects during SEM imaging, limiting the feasibility of obtaining high-quality images; the white scale bars in the images correspond to 100 nm).

SUPPORTING INFORMATION

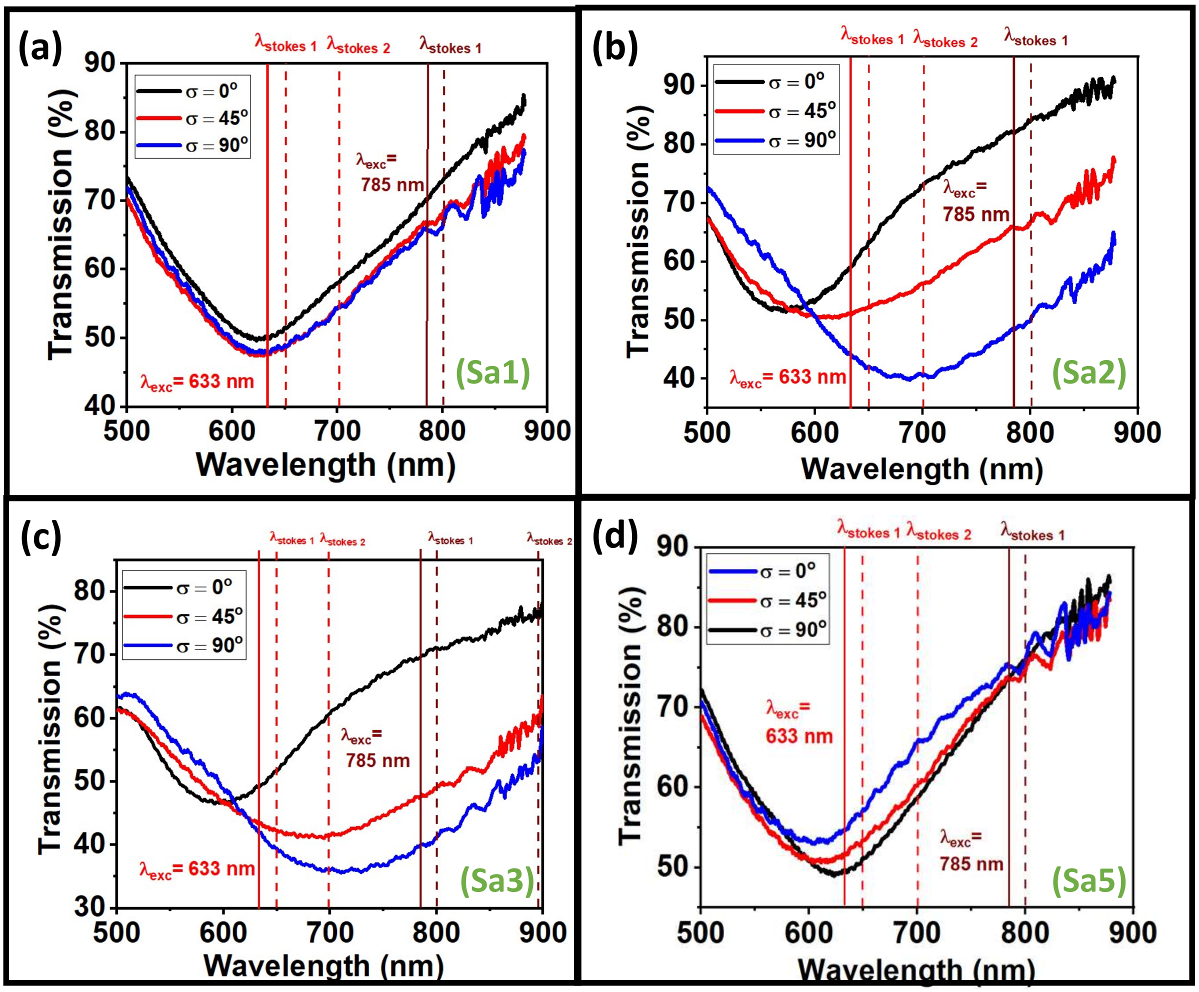

Figure S5. Polarized transmission spectra measured for σ = 0° 45°and 90° of the samples: (**a**) Sa1(flat template), **(b)** Sa2($P \cong d$), (**c**) Sa3($P \cong d$) and (**d**) Sa5($P >> d$). The vertical lines indicate the laser λ_{exc} and its corresponding λ_{Stokes} positions on the spectra.

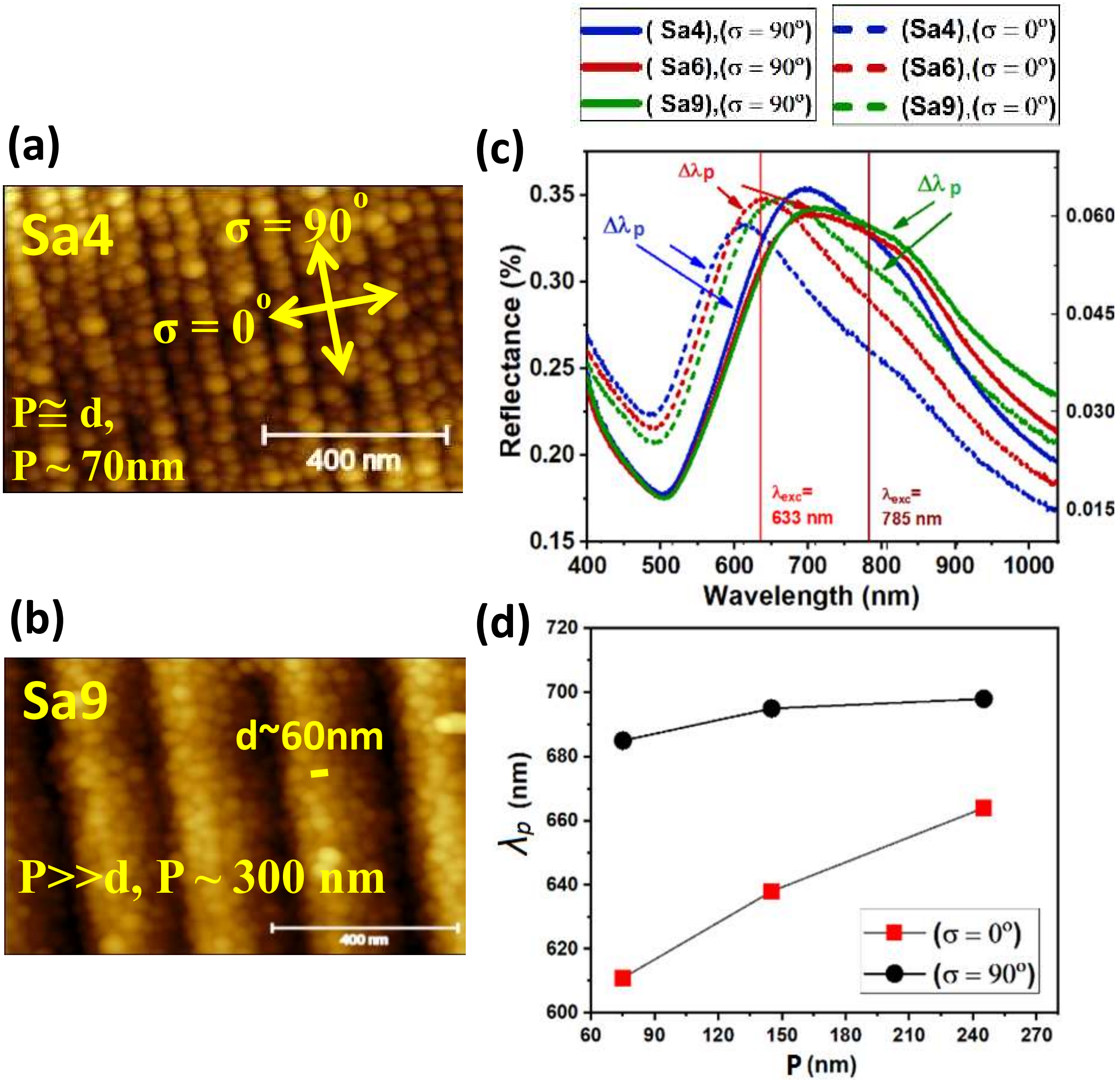

Figure S6. AFM topography images of samples (**a**) Sa4 and (**b**) Sa9, **(c)** reflectance spectra of samples Sa4, Sa6 and Sa9. (**d**) comparison of the position of λ_p of samples Sa4, Sa6 and Sa9 listed in Figure S5b. showing the largest value of λ_p for the samples with the shortest corrugation period *P*.

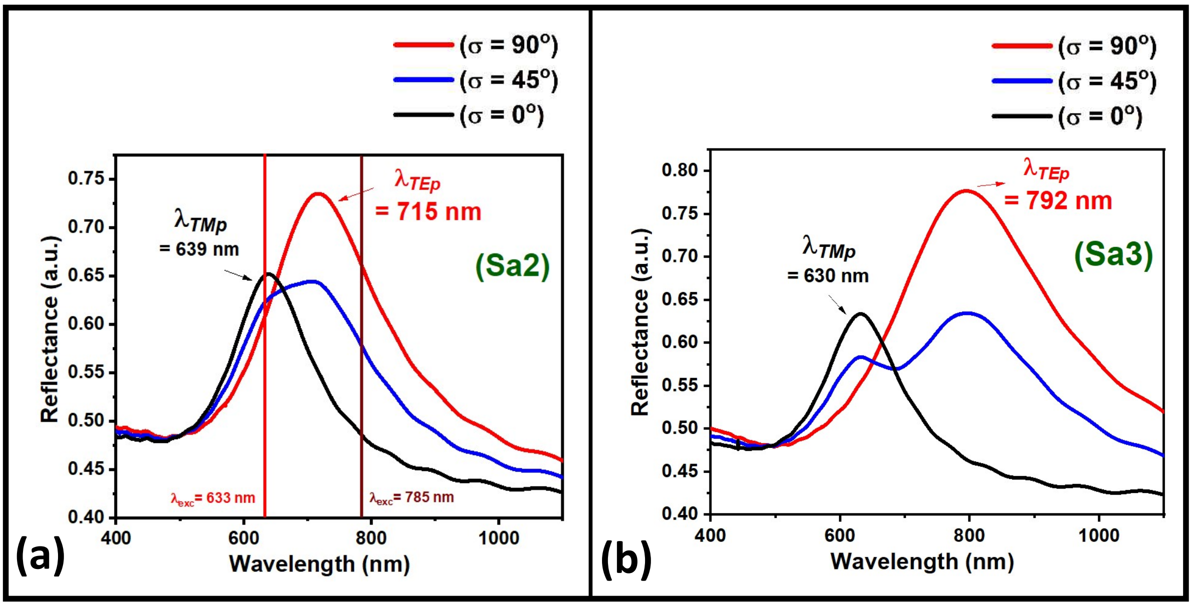

Figure S7. Simulated polarized reflectance spectra for angles θ=50˚, *σ* = 0˚, 45˚ and 90˚ for samples (**a**) Sa2 (spherical) and (**b**) Sa3 (elliptical) Au grains.

SUPPORTING INFORMATION

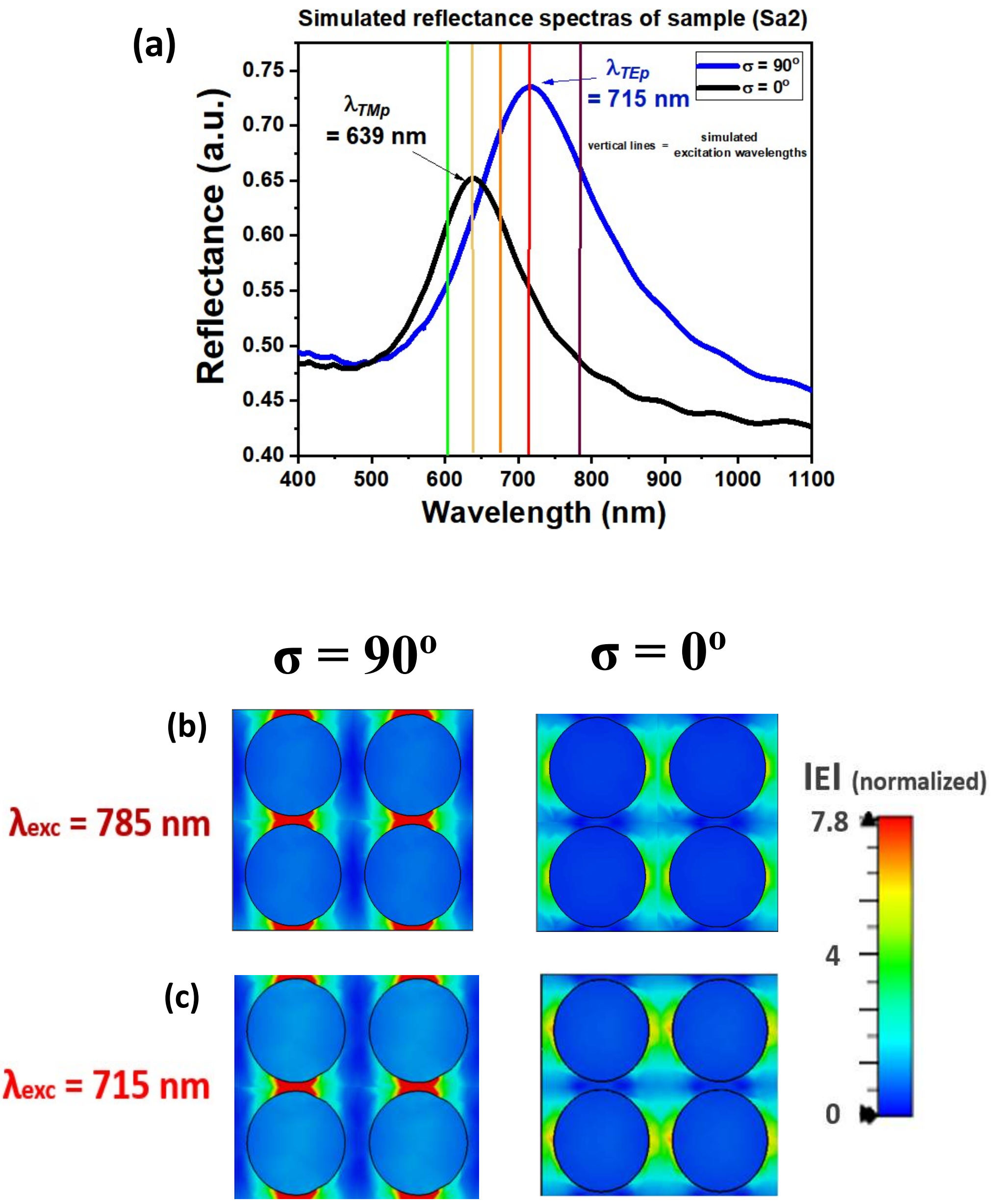

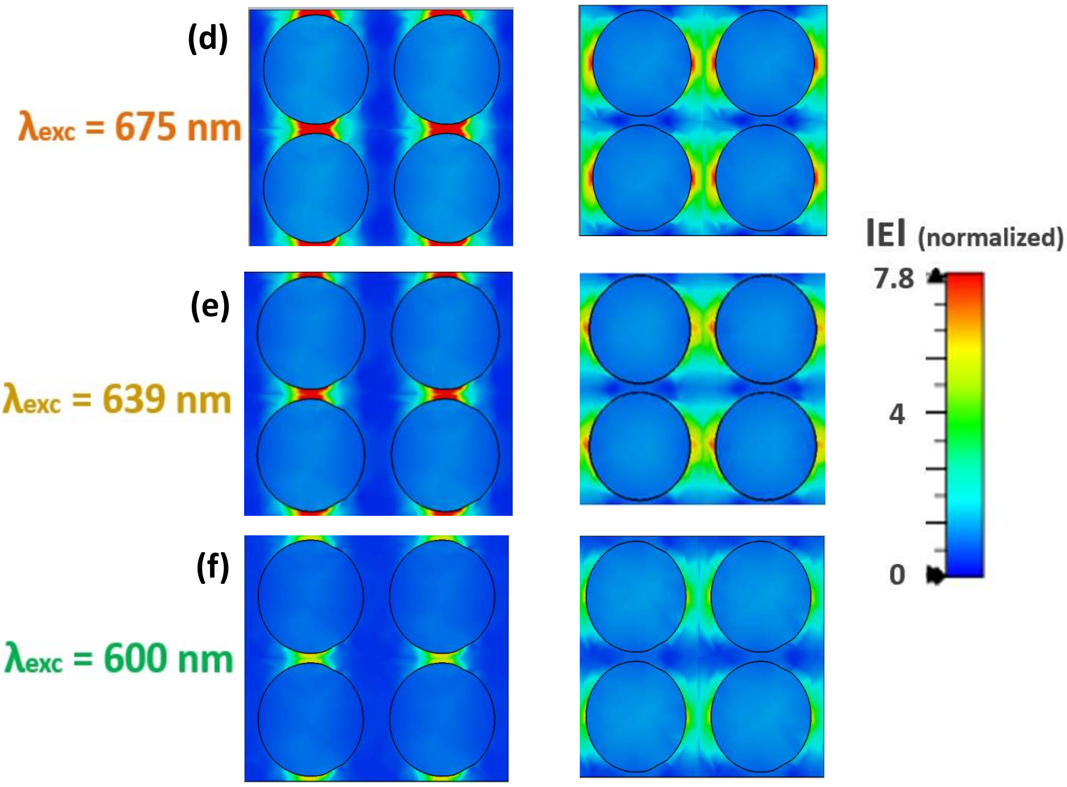

Figure S8. (**a)** Simulated polarized reflectance spectra of the homogenized sample (Sa2) at (σ = 0 ˚) and (σ = 90 ˚) the vertical lines in the spectra represents the excitation wavelength positions for the simulated near-field distribution images in (**b)** for λ_{exc} = 785 nm, (**c)** for λ_{exc} = 715 nm, (**d)** for λ_{exc} = 675 nm, (**e)** for λ_{exc} = 639 nm and (**f)** for λ_{exc} = 600 nm. (left column represent near-field distribution images at (σ = 90˚) and right at (σ = 0˚). 'The near-field results are presented for a (2 X 2) cell fragment of the periodic, in-plane infinite structure.

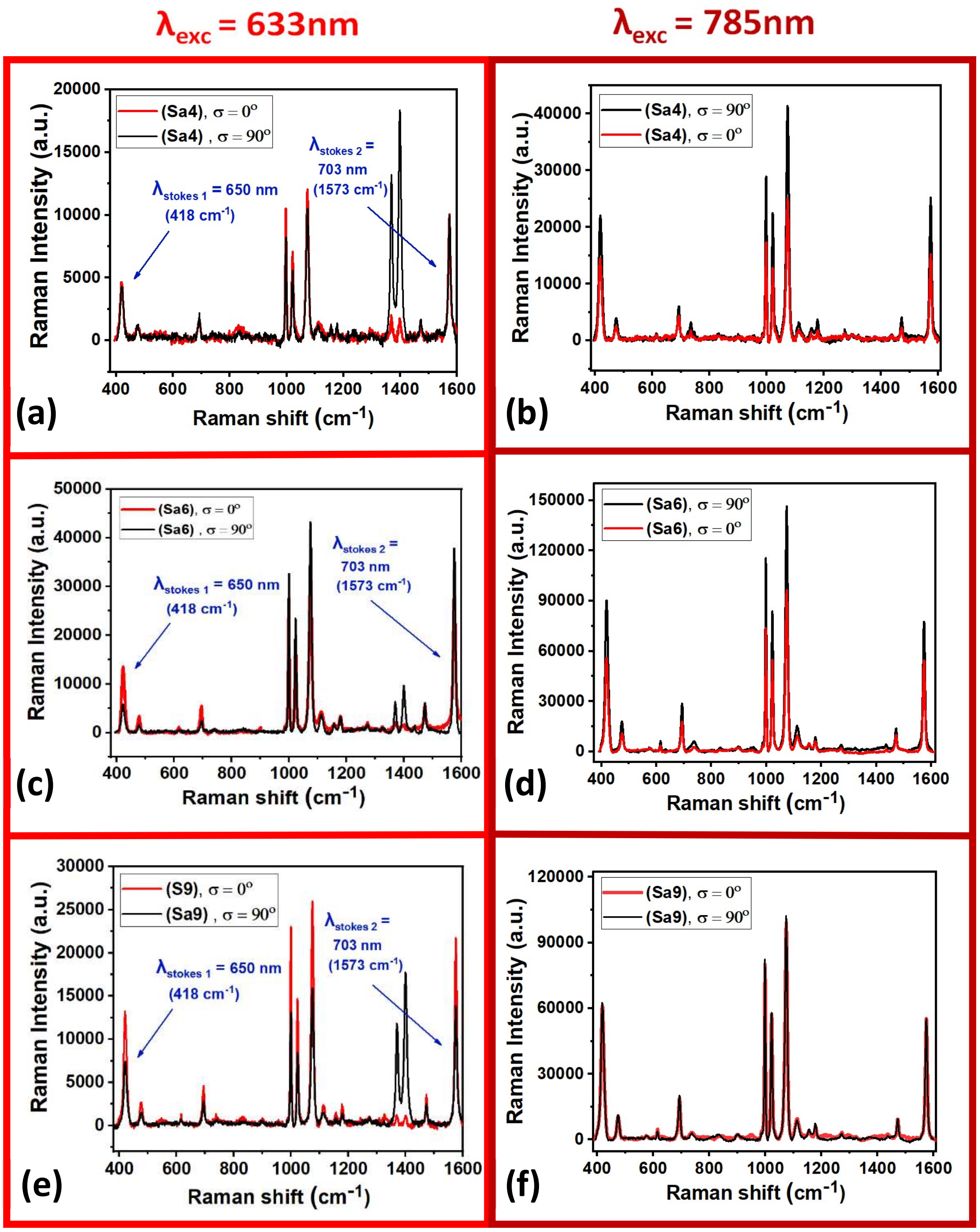

Figure S9. SERS spectra measured at λ_{exc} = 633 nm for the samples (**a)** Sa4, (**c)** Sa6 and (**e)** Sa9 at both σ = 0° and 90° polarizations. (left column). SERS spectra measured at λ_{exc} = 785 nm for the (**b)** Sa4, (**d)** Sa6 and (**f)** Sa9 at both σ = $^{\circ}$ and 90° polarizations. (right column).

SUPPORTING INFORMATION

Experimental results similar to the ones observed for sample Sa3 were also obtained for sample Sa6 as shown in Figure S7c. where its resonance position λ_{TMp} along $\sigma = 0^\circ$ in Figure S5c. is closer to the λ_{exc} = 633 nm and its corresponding $\lambda_{Stokes\ 1}$ when compared to its $\lambda_{Stokes\ 2}$. Thus this sample Sa6 exhibits trends in Figure S7c. similar to what was observed for sample Sa3.

SUPPORTING INFORMATION

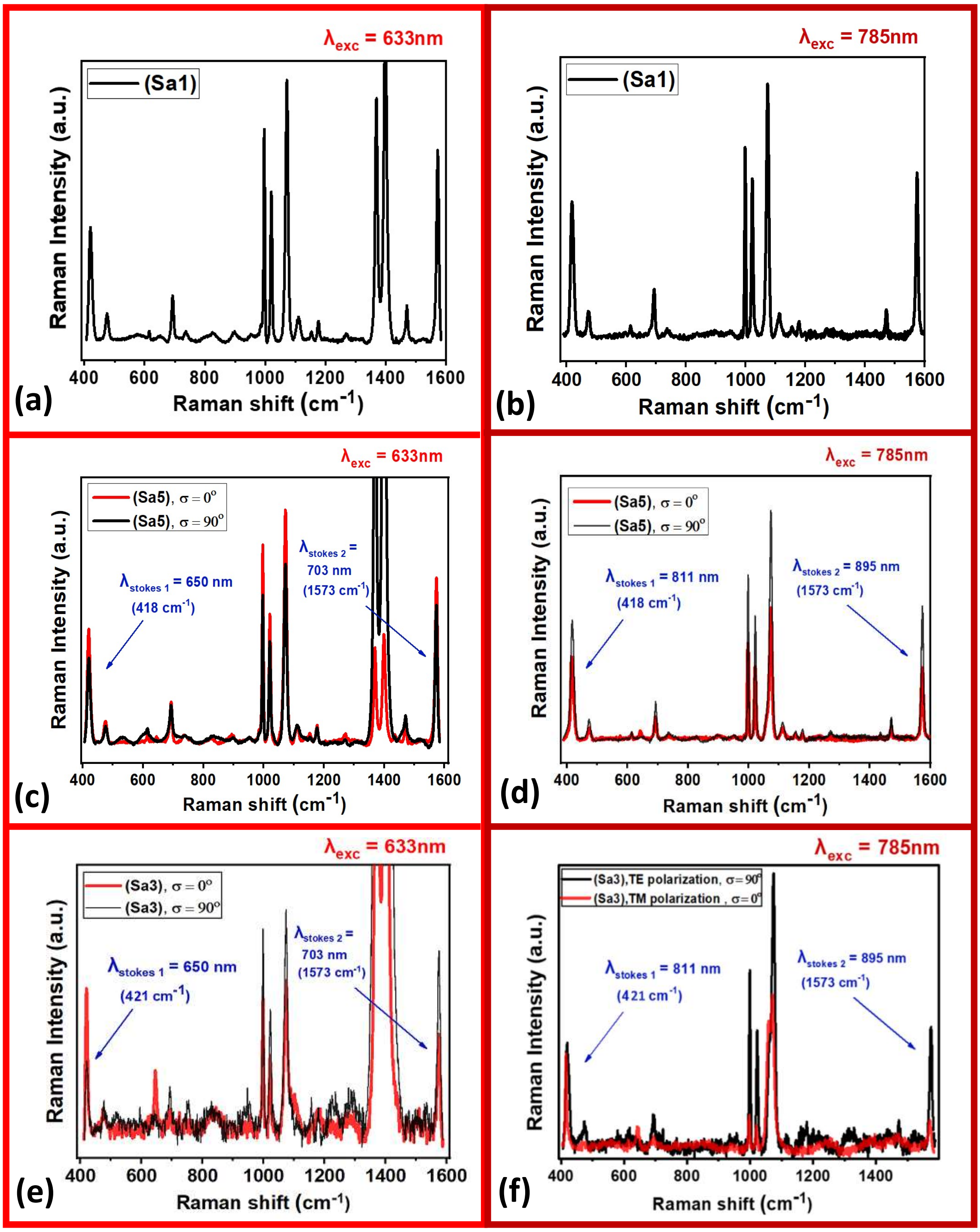

Figure S10. SERS spectra measured at λ_{exc} = 633 nm for the samples (**a**) Sa1, (**c**) Sa5 and (**e**) Sa3 at both σ = 0° and 90° polarizations (left column). SERS spectra measured at λ_{exc} = 785 nm for the samples (**b**) Sa1, (**d**) Sa5 and (**f**) Sa3 at both σ = 0° and 90° polarizations (right column).

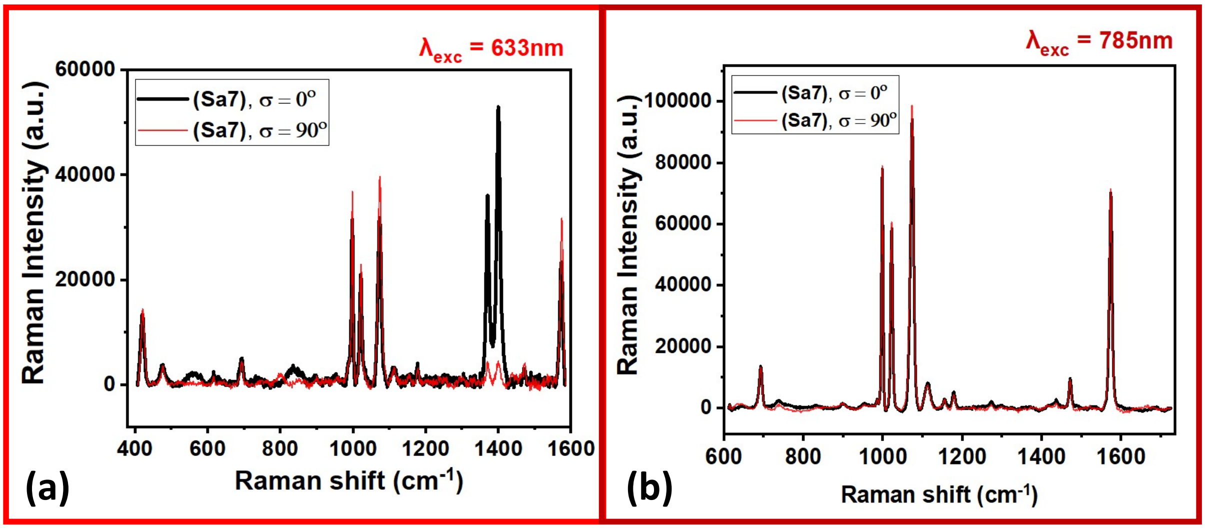

Figure S11. SERS spectra measured at (**a**) λ_{exc} = 633 nm for the sample Sa7 at both σ = 0° and 90° polarizations. (**b**) at λ_{exc} = 785 nm for the samples Sa4 at both σ = 0° and 90° polarizations.

Furthermore, sample Sa7 coated with a thicker gold layer (t ~ 10 nm) shows that its λ_{TMp} position along σ = 0° is red-shifted relative to λ_{exc} = 633 nm in Figure 1d, and thus a reversed SERS dichroism is observed (in Figure S10.) where both $\lambda_{Stokes\ 1}$ and $\lambda_{Stokes\ 2}$ peaks exhibits a $cos^2(\sigma)$ dependency.

SERS Mapping

The SERS mapping measurements were performed in the fabricated Au array samples with optical properties presented in Figure S12.

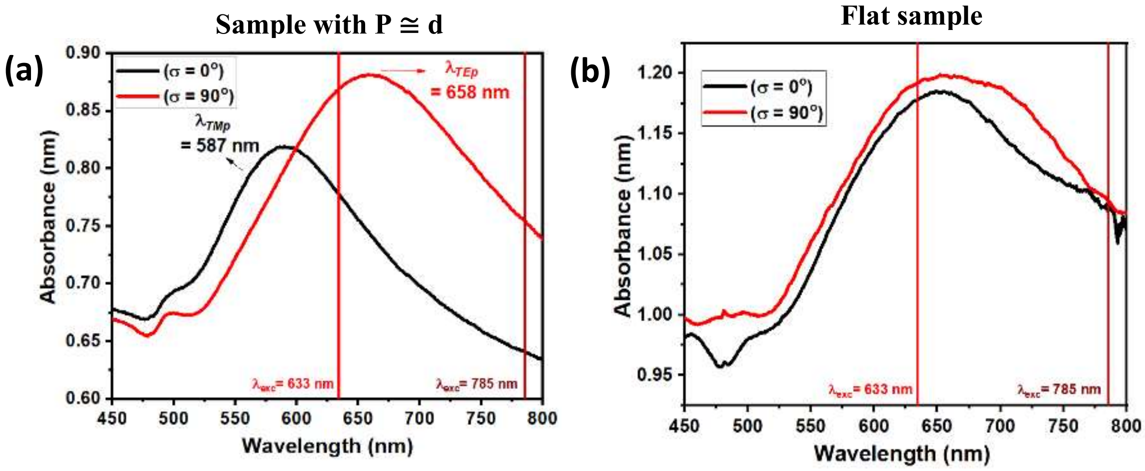

Figure S12. (a) Polarized reflectance spectra for sample with P ≅ d exhibiting anisotropic plasmon resonance peaks at λ_{TMp} ~ 587 nm at σ = 0 ˚and λ_{TEp} ~ 658 nm at σ = 90 ˚**(b)** Polarized reflectance spectra for a flat sample exhibiting isotropic plasmon resonance peaks at λ_{TMp} ~ 660 nm at σ = 0 ˚and λ_{TEp} ~ 660 nm at σ = 90 ˚. *Vertical lines in the spectra indicates the experimental excitation wavelength (λ_{exc}) in which SERS mappings was performed for different polarizations and wavelengths.*

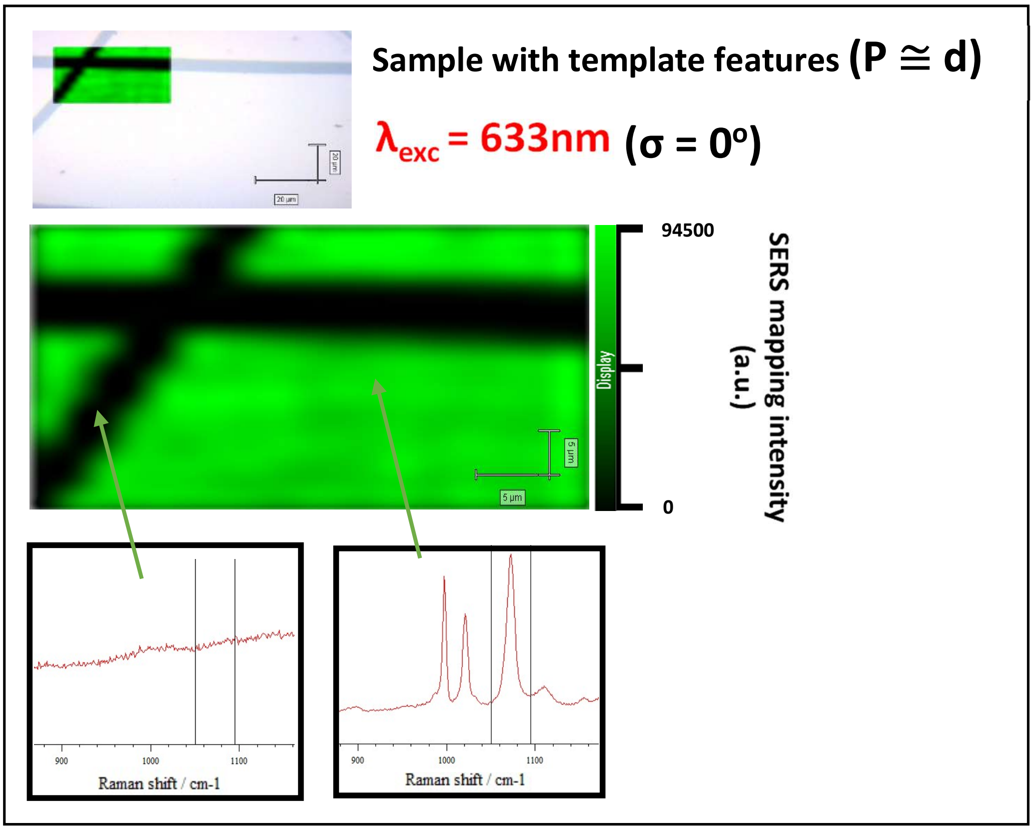
Sample with template features (P ≅ d)
λexc = 633nm (σ = 0º)
94500
0
SERS mapping intensity (a.u.)
Display
5 µm
20 µm
900
1000
1100
Raman shift / cm-1

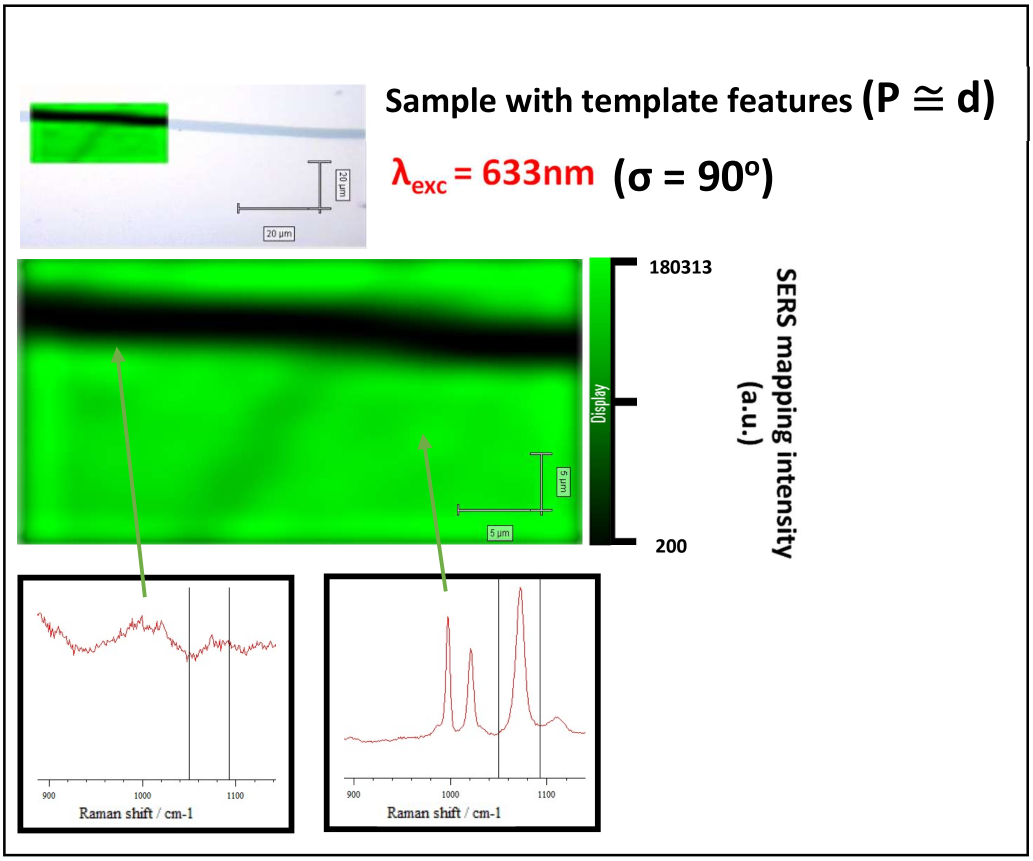
Sample with template features (P ≅ d)
λexc = 633nm (σ = 90º)
180313
200
SERS mapping intensity (a.u.)
Display
5 µm
20 µm
900
1000
1100
Raman shift / cm-1

Figure S13. SERS mapping measurements of phenyl thiolate peak 1081 cm^{-1} under λ_{exc} = 633 nm on sample with P ≅ d exhibiting anisotropic SERS and long-ranged uniform sensing capability of the samples (the dark line in the SERS image represent a scratch on the Au layer).

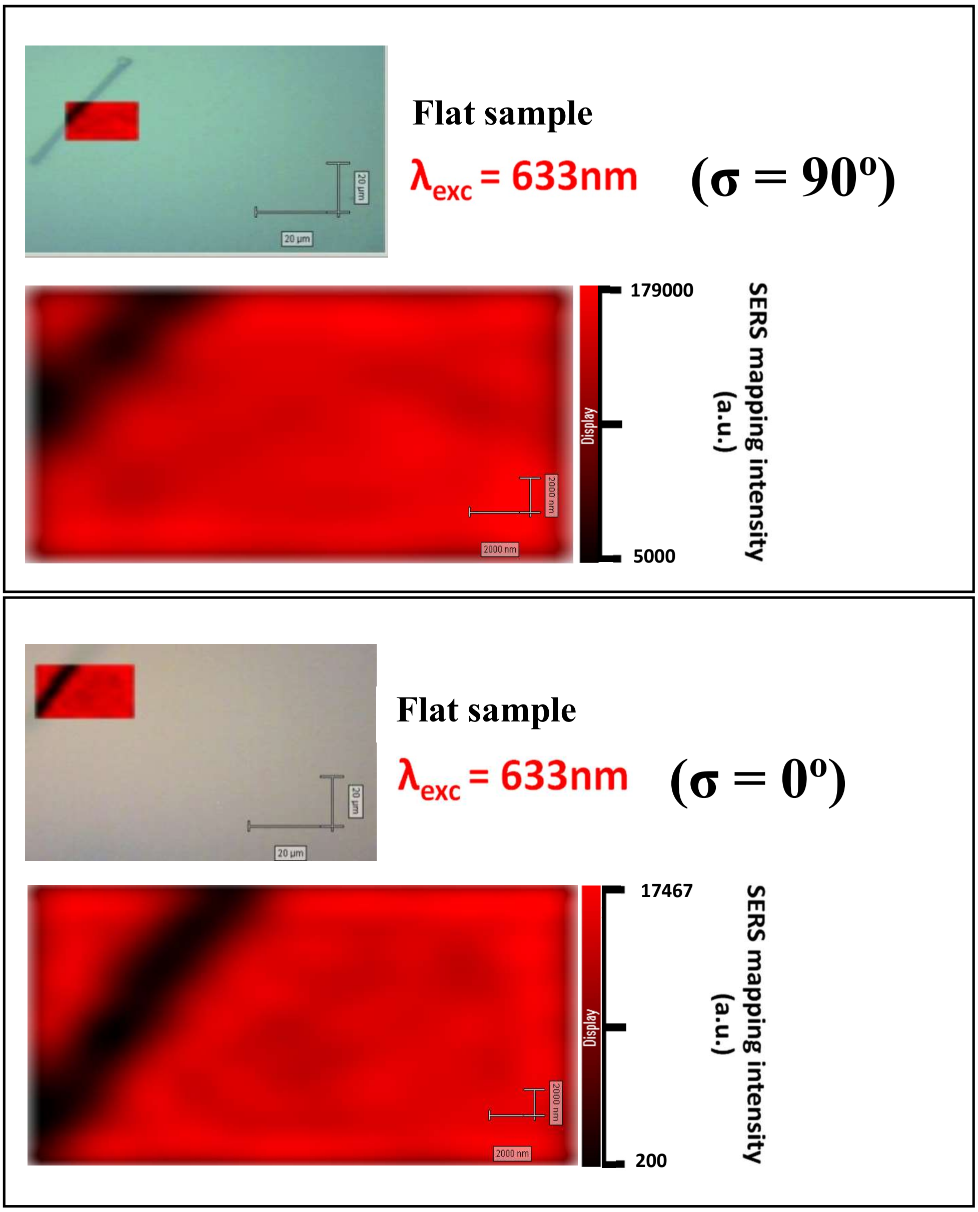

Figure S14. SERS mapping measurements of phenyl thiolate peak 1081 cm-1 under λ_{exc} = 633 nm on the flat sample in the exhibiting isotropic and long-ranged uniform sensing capability of the samples (the dark line in the SERS image represent a scratch on the Au layer).

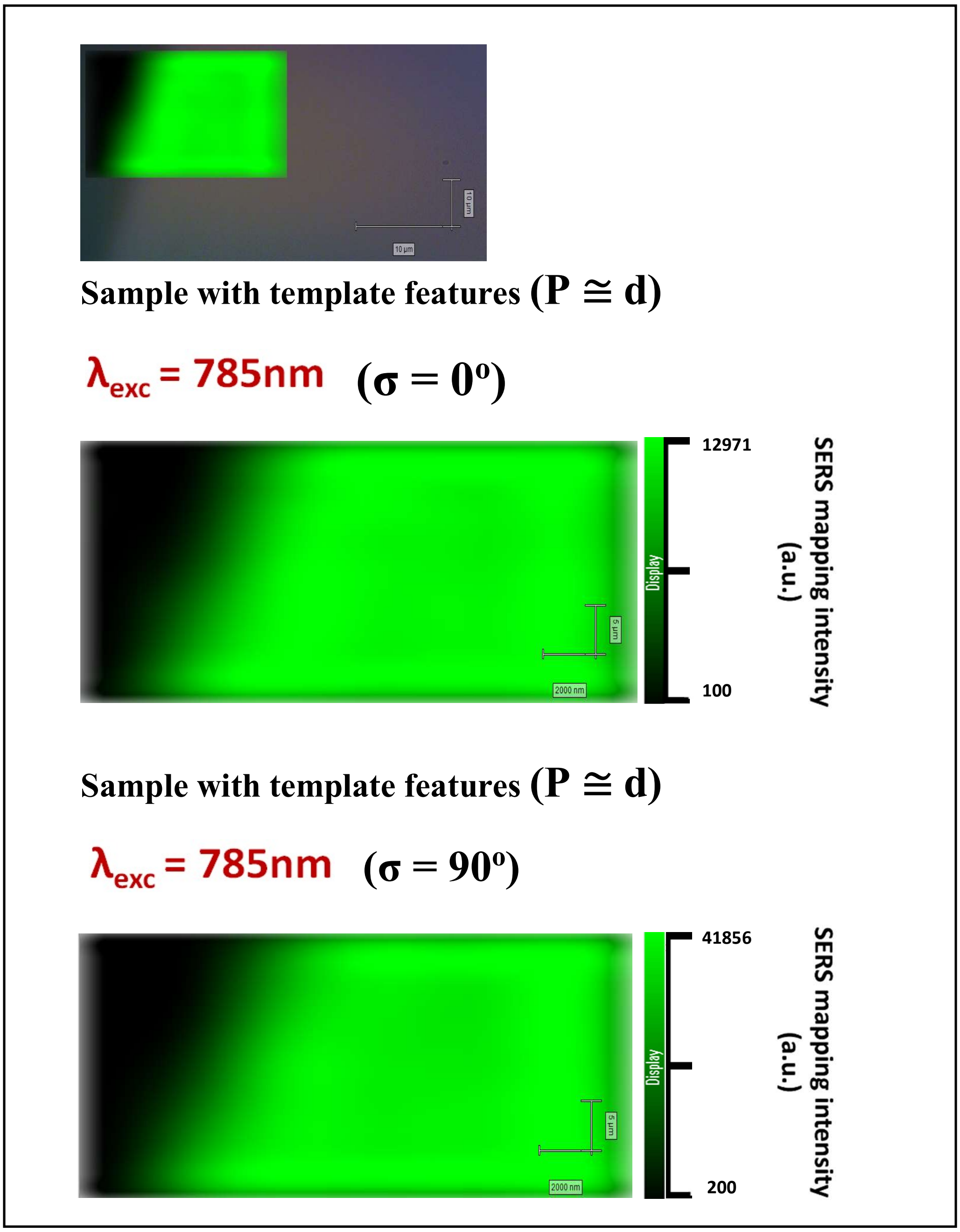

Figure S15. SERS mapping measurements of phenyl thiolate peak 1081 cm-1 under λ_{exc} = 785 nm on sample with P ≅ d in the exhibiting anisotropic and long-ranged uniform sensing capability of the samples (the dark line in the SERS image represent a scratch on the Au layer).

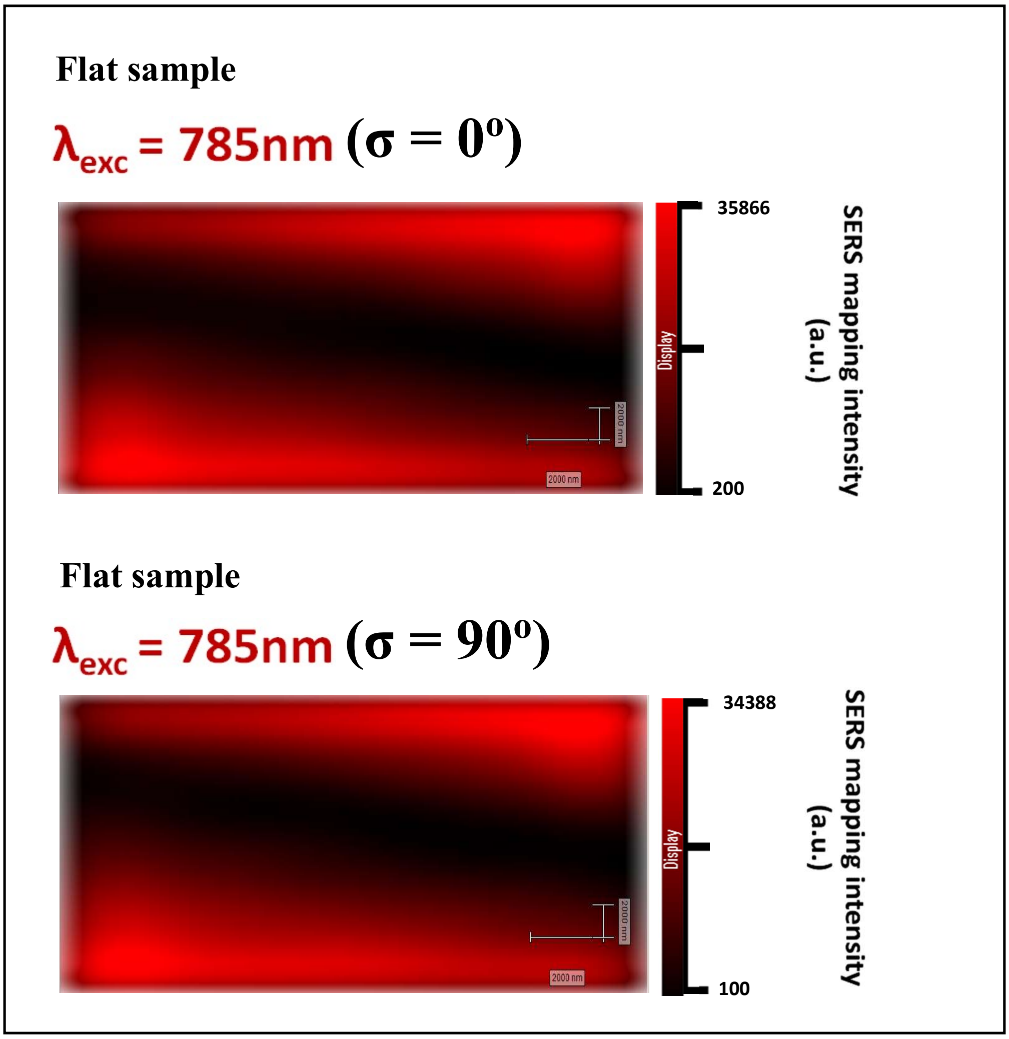

Figure S16. SERS mapping measurements of phenyl thiolate peak 1081 cm-1 under λ_{exc} = 785 nm on the flat sample in the exhibiting isotropic and long-ranged uniform sensing capability of the samples (the dark line in the SERS image represent a scratch on the Au layer).

SUPPORTING INFORMATION

SERS enhancement calculation

Calculation of enhancement factor for the phenyl thiolate peak 1081 cm^{-1} on the samples Sa2 and Sa6 with template features P ≅ d. The sample Sa2 has Au grain diameter of d~27 nm and Sa6 has d~30 nm.

The enhancement factors were calculated for excitations under λ_{exc} = 633 and 785 nm wavelength at both σ = 90 ˚and σ = 0 ˚(related to the super and sub-radiant collective plasmon modes frequencies).

The enhancement factor was calculated based on the formula[4,5].

$$\text{Enhancement Factor (EF)}=\frac{I_{SERS}}{N_{SERS}} \times \frac{N_{ref}}{I_{ref}}$$

Where, $\mathbf{I_{SERS}}$ = intensity of the SERS, $\mathbf{I_{ref}}$ = intensity of reference Raman, $\mathbf{N_{SERS}}$ and $\mathbf{N_{ref}}$ = no of phenyl thiolate molecules in the illuminated volume.

Thio-phenol dissolved in an aqueous solution as 0.0097 M was measured for I_{ref} values under both λ_{exc} = 633 and 785 nm excitation wavelength with a 50x, 0.7 N.A. numerical aperture objective.

The minimum laser beam waist diameter ω can be calculated from the formula

$$\omega = \frac{(4*\lambda)}{\pi*\text{NA}}$$

where, λ is the laser wavelength and NA is the numerical aperture of the focusing objective.

$$\omega_{633nm} = 1.152\ \mu m$$

$$\omega_{785nm} = 1.42\ \mu m$$

The depth of focus $z = \frac{2\pi*\omega^2}{\lambda}$

$$Z_{633nm} = 13.1\ \mu m$$

$$Z_{785nm} = 16.1\ \mu m$$

The focal volume of the illumination is $V_F = (\frac{\pi}{2})^{1.5} * \omega^2 * z$

$$V_{F\ 633nm} = 34.2\ \mu m^3$$

$$V_{F\ 785nm} = 63.8\ \mu m^3$$

SUPPORTING INFORMATION

The number of thio-phenol molecules in the N_{ref} is $N_{ref} = V_F * C_{Thiophenol} * N_A$ given by

$N_{ref\ 633nm} = 1.9x10^8$

$N_{ref\ 785nm} = 3.7x10^8$

where, $C_{Thiophenol}$ is the molar concentration of the thio-phenol dissolved in the aqueous solution, and N_A is the Avogadro number.

The thio-phenol molecules chemisorbs as a monolayer onto the Au surface as phenyl-thiolate.

The area of laser spot is $A_{laser} = \pi(^{\omega}/_{2})^2$

The Au array contained 20% less Au surface area when compared to the percolated laterally continuous flat Au surface.

$A_{laser\ 633nm} = 1.04\ \mu m^2$

$A_{laser\ 785nm} = 1.58\ \mu m^2$

The occupied surface area $A_{thiophenol}$ for a single thiophenol molecule[6].

$A_{thiophenol} = 1.16 \times 10^{-16}\ m^2$

The number of thiophenol molecules in the SERS measurements is $N_{SERS} = {}^{A_{laser}}/_{A_{Thiophenol}}$

$N_{SERS\ 633nm} = 7.2x10^3$

$N_{SERS\ 785nm} = 1.08x10^4$

SUPPORTING INFORMATION

Table S2. Calculated parameters to obtain enhancement factor for sample **Sa2** with template features P ≅ d and Au grain diameter d~27 nm.

	λ_{exc} = 633 nm, σ = 0 °	λ_{exc} = 633 nm, σ = 90 °	λ_{exc} = 785 nm, σ = 0 °	λ_{exc} = 785 nm, σ = 90 °
I_{SERS}	65384	85004	14800	63163
N_{SERS}	$7.2x10^3$	$7.2x10^3$	$1.08x10^4$	$1.08x10^4$
I_{ref}	544	544	763	763
N_{ref}	$1.9x10^8$	$1.9x10^8$	$3.7x10^8$	$3.7x10^8$

Enhancement factor (EF) of Sample (Sa2)

$$EF_{\lambda exc\ 633nm,\ \sigma = 0°} = 4.1x10^6$$

$$EF_{\lambda exc\ 633nm,\ \sigma = 90°} = 3.6\ x10^6$$

$$EF_{\lambda exc\ 785nm,\ \sigma = 0°} = 2.8\ x10^6$$

$$EF_{\lambda exc\ 785nm,\ \sigma = 90°} = 6.6\ x10^5$$

Table S3. Calculated parameters to obtain enhancement factor for sample **Sa6** with template features P ≅ d and Au grain diameter d~60nm.

	λ_{exc} = 633 nm, σ = 0 °	λ_{exc} = 633 nm, σ = 90 °	λ_{exc} = 785 nm, σ = 0 °	λ_{exc} = 785 nm, σ = 90 °
I_{SERS}	36787	43926	96557	148052
N_{SERS}	$7.2x10^3$	$7.2x10^3$	$1.08x10^4$	$1.08x10^4$
I_{ref}	544	544	763	763
N_{ref}	$1.9x10^8$	$1.9x10^8$	$3.7x10^8$	$3.7x10^8$

Enhancement factor (EF) of Sample (Sa6)

$$EF_{\lambda exc\ 633nm,\ \sigma = 0°} = 1.7x10^6$$

$$EF_{\lambda exc\ 633nm,\ \sigma = 90°} = 2.1x10^6$$

$$EF_{\lambda exc\ 785nm,\ \sigma = 0°} = 4.3\ x10^6$$

$$EF_{\lambda exc\ 785nm,\ \sigma = 90°} =6.6\ x10^6$$

SUPPORTING INFORMATION

References

(1) Hedl, E.; Bregović, V. B.; Rakić, I. Š.; Mandić, Š.; Samec, Ž.; Bergmann, A.; Sancho-Parramon, J. Optical Properties of Annealed Nearly Percolated Au Thin Films. *Opt Mater (Amst)* 2023, *135*. https://doi.org/10.1016/j.optmat.2022.113237.

(2) Maniyara, R. A.; Rodrigo, D.; Yu, R.; Canet-Ferrer, J.; Ghosh, D. S.; Yongsunthon, R.; Baker, D. E.; Rezikyan, A.; García de Abajo, F. J.; Pruneri, V. Tunable Plasmons in Ultrathin Metal Films. *Nature Photonics*. Nature Publishing Group May 1, 2019, pp 328–333. https://doi.org/10.1038/s41566-019-0366-x.

(3) Gabai, R.; Ismach, A.; Joselevich, E. Nanofacet Lithography: A New Bottom-up Approach to Nanopatterning and Nanofabrication by Soft Replication of Spontaneously Faceted Crystal Surfaces. *Advanced Materials* 2007, *19* (10), 1325–1330. https://doi.org/10.1002/adma.200601625.

(4) Liu, K.-K.; Tadepalli, S.; Tian, L.; Singamaneni, S., Size-Dependent Surface Enhanced Raman Scattering Activity of Plasmonic Nanorattles. *Chemistry of Materials* 2015, *27* (15), 5261-5270. https://doi.org/10.1021/acs.chemmater.5b01401.

(5) 2. Stiles, P. L.; Dieringer, J. A.; Shah, N. C.; Van Duyne, R. P., Surface-Ehanced Raman Spectroscopy. *Annu. Rev. Ana.l Chem.* (Palo Alto Calif) 2008, *1*, 601-26. https://doi.org/10.1146/annurev.anchem.1.031207.112814.

(6) Gui, J. Y.; Stern, D. A.; Frank, D. G.; Lu, F.; Zapien, D. C.; Hubbard, A. T. Adsorption and Surface Structural Chemistry of Thiophenol, Benzyl Mercaptan, and Alkyl Mercaptans. Comparative Studies at Ag(Lll) and Pt(Lll) Electrodes by Means of Auger Spectroscopy, Electron Energy Loss Spectroscopy, Low-Energy Electron Diffraction, and Electrochemistry; 1991; Vol. 7. https://pubs.acs.org/sharingguidelines.
